# QTL analysis and fine mapping of a QTL for yield-related traits in wheat grown in dry and hot environments

**DOI:** 10.1007/s00122-019-03454-6

**Published:** 2019-10-04

**Authors:** Habtamu Tura, James Edwards, Vijay Gahlaut, Melissa Garcia, Beata Sznajder, Ute Baumann, Fahimeh Shahinnia, Matthew Reynolds, Peter Langridge, Harindra Singh Balyan, Pushpendra K. Gupta, Thorsten Schnurbusch, Delphine Fleury

**Affiliations:** 1 School of Agriculture, Food and Wine, Waite Campus, University of Adelaide, PMB1, Glen Osmond, SA 5064 Australia; 2 Australian Grain Technologies, 20 Leitch Road, Roseworthy, SA Australia; 3 Department of Genetics and Plant Breeding, Ch. Charan Singh University, Meerut, India; 4 Present Address: Institute for Crop Science and Plant Breeding, Bavarian State Research Center for Agriculture, Am Gereuth 8, 85354 Freising, Germany; 5 International Maize and Wheat Improvement Center (CIMMYT), Int. AP 6-641, 06600 Mexico, D.F. Mexico; 6 Julius-Kühn-Institute, Königin-Louise-Str 19, 14195 Berlin, Germany; 7 Present Address: Leibniz-Institute of Plant Genetics and Crop Plant Research (IPK), Corrensstr. 3, 06466 Gatersleben, Germany

## Abstract

Genetic control of grain yield and phenology was examined in the Excalibur/Kukri doubled haploid mapping population grown in 32 field experiments across the climatic zones of southern Australia, India and north-western Mexico where the wheat crop experiences drought and heat stress. A total of 128 QTL were identified for four traits: grain yield, thousand grain weight (TGW), days to heading and grain filling duration. These QTL included 24 QTL for yield and 27 for TGW, showing significant interactions with the environment (*Q* * *E*). We also identified 14 QTL with a significant, small main effects on yield across environments. The study focussed on a region of chromosome 1B where two main effect QTL were found for yield and TGW without the confounding effect of phenology. Excalibur was the source of favourable alleles: *QYld.aww*-*1B.2* with a peak at 149.5–150.1 cM and *QTgw.aww*-*1B* at 168.5–171.4 cM. We developed near isogenic lines (NIL) for the interval including *QYld.aww*-*1B.2* and *QTgw.aww*-*1B* and evaluated them under semi-controlled conditions. Significant differences in four pairs of NIL were observed for grain yield but not for TGW, confirming a positive effect of the Excalibur allele for *QYld.aww*-*1B.2*. The interval containing *QYld.aww*-*1B.2* was narrowed down to 2.9 cM which corresponded to a 2.2 Mbp genomic region on the chromosome 1B genomic reference sequence of cv. Chinese Spring and contained 39 predicted genes.

**Electronic supplementary material:**

The online version of this article (10.1007/s00122-019-03454-6) contains supplementary material, which is available to authorized users.

## Introduction

Wheat is an important crop worldwide and is grown on more than 51%, 28% and 10% of the total area under cereal cultivation in Australia, India and Mexico, respectively (http://faostat3.fao.org/). The cropping area affected by drought and heat stress is increasing due to climate change, population pressure and deforestation. Drought affects at least 60 million hectares of wheat area (Braun et al. 2010; Monneveux et al. 2012) causing more than 50% loss in wheat yield globally (Bray et al. 2000; Kosina et al. 2007; Nezhad et al. 2012). These losses due to drought account for 3.5 million tonnes annual losses in Australia, thus contributing to the country’s low average yields of around 1.7 tonnes/ha (Gavran 2012; Ray et al. 2013). In South Australia, cyclic drought, characterized by sporadic rainfall during anthesis and grain filling, can cause total crop loss under extreme drought conditions (Izanloo et al. 2008).

Grain yield is a primary target for wheat breeding programs globally. Yield is the result of cumulative effects of many traits and processes in the plant that interact with each other and the environment throughout the plant growth period. The development of high yielding varieties for areas with limited water supply is hampered by the complex polygenic nature of the trait, its low heritability and large genotype * environment (*G* * *E*) interactions (Blum 2011; Passioura 2012). Most traits that are associated with drought tolerance including transpiration efficiency (Condon et al. 1993; Nakhforoosh et al. 2016; Rebetzke et al. 2002), leaf rolling, carbohydrate storage and remobilization (Ovenden et al. 2017; Rebetzke et al. 2008; Saint Pierre et al. 2010), osmotic adjustment (Abdolshahi et al. 2015; Morgan 2000) and canopy temperature (Blum et al. 1982; Reynolds et al. 1998; Pinto et al. 2010) vary across sites and years. Drought is also often concomitant with heat stress in the field.

Improvement in a complex trait such as yield for dry and hot areas can be facilitated through quantitative trait loci (QTL) mapping and QTL cloning. This involves identification of QTL for yield in targeted environments, fine mapping of QTL and finding molecular markers tightly linked to the trait QTL for molecular breeding (Graziani et al. 2014; Soriano et al. 2017). QTL for grain yield and yield components in wheat under drought and heat have been reported in many studies (Pinto et al. 2010; Maccaferri et al. 2010; McIntyre et al. 2010; Bennett et al. 2012a, b; Graziani et al. 2014; Shukla et al. 2015; also see review by Tricker et al. 2018), but none of these QTL have been cloned in wheat. In addition to the complex quantitative nature of yield, the lack of a reference wheat genome sequence has been an obstacle for QTL cloning until recently. The International Wheat Genome Sequence Consortium (IWGSC) released a wheat genome reference sequence along with annotated genes (called RefSeq v.1.0, IWGSC 2018) which is now publicly available at http://wheaturgi.versailles.inra.fr/Seq-Repository. This information and the whole genome sequence dataset of 16 wheat varieties (Edwards et al. 2012) enable the identification of new single nucleotide polymorphism (SNP) markers for QTL fine mapping, thus facilitating the development of functional markers/genes to be used for improvement in yield under drought (Watson-Haigh et al. 2018).

The objectives of the present study were to (i) identify yield and TGW QTL in Excalibur/Kukri doubled haploid (DH) population under dry and hot environments on three continents, (ii) fine map a QTL for yield under severe drought and heat on chromosome 1B using NIL and (iii) identify predicted genes in the genomic interval containing the yield QTL.

## Materials and methods

### Plant materials

A DH population, consisting of 233 lines derived from a cross between two Australian spring wheat cultivars, Excalibur and Kukri, was used for QTL mapping. The parental line Excalibur (RAC177/‘Monoculm’//RAC311S) is a drought-tolerant cultivar that was released by the University of Adelaide in 1991. Kukri (76ECN44/76ECN36//RAC549; MADDEN/6*RAC177) is a drought-sensitive cultivar released by the University of Adelaide in 1999. The two cultivars have the *Rht*-*D1b* semi-dwarfing allele, and although they segregate for the vernalization gene *Vrn*-*A1*, they have similar phenology (Izanloo et al. 2008; Asif et al. 2018). Both cultivars also have high-yield potential in non-stressed environments (Izanloo et al. 2008).

Five pairs of NIL (EK428_2, EK428_8, EK570, EK405 and EK664) were developed from single plant, heterozygous for the interval containing *QYld.aww*-*1B.2* and *Qtgw.aww*-*1B* following the method described by Tuinstra et al. (1997). Five Excalibur/Kukri RIL, heterozygous at the markers BS00066864 and adw572, were selected from a collection of ~ 2000 Excalibur/Kuri RIL (F2:5) using LGC Genomics Kompetitive Allele Specific PCR (KASP™) assays. Eight seeds of each of the five RIL (F2:6) were sown in September 2015 at Urrbrae, SA. Single plant DNA was assayed with four markers (BS00066864, adw1218477, BS00084985, adw572) and heterozygous F2:6 plants were self-fertilized. Segregating progenies (F2:7 plants) were genotyped with the four markers above; homozygous plants carrying Excalibur or Kukri allele were selected as NIL (Table S1). The F_2:8_ NIL were seed multiplied in a greenhouse from March to June 2016 and genotyped with 22 KASP™ markers to verify whether the genetic background was fixed (Table S1).

### Genetic map of DH population

DNA preparation, genotyping and construction of the genetic map of the Excalibur/Kukri DH population were described earlier (Asif et al. 2018). The map construction and diagnostics were performed with functions and workflow of the R package ASMap (Taylor and Butler 2017) and R/qtl available in the R Statistical Computing Environment (R Core Team 2016). The linkage map was based on 155 lines and contained 3502 markers including 174 simple sequence repeat (SSR), 285 diversity arrays technologies (DArT) markers, 2970 genotyping-by-sequencing (GBS) markers, 51 SNP, 18 insertion site-based polymorphism (ISBP) and 5 gene-based markers (*glutenin B1, Grain Weight 2 TaGW2, Sr15/Lr20, Vernalization Vrn*-*A1*) (Asif et al. 2018). The markers were assembled into 28 linkage groups and assigned to 21 wheat chromosomes. The total length of the genetic map was 2765 cM, containing 849 unique loci with an average distance of 3 cM (min = 0.1 and max = 33 cM) between two adjacent markers. For QTL analysis, six additional DH lines were used, which were set aside during construction of genetic map due to the level of missing value > 20% in the raw data of un-ordered markers; the missing genotypic data for mapped markers were imputed using available linkage map, as described in the next section.

### Chromosome 1B high-resolution genetic map

In order to increase the marker density on genetic map of chromosome 1B, we used five sources of genome sequences for marker selection and design: (i) the 90K Wheat Illumina Infinium iSelect genotyping array (Wang et al. 2014), (ii) GBS markers (Asif et al. 2018), (iii) the Avalon/Cadenza chromosome 1B genetic map (Allen et al. 2013), (iv) the Breeders’ 35K Axiom^®^ array (Wilkinson et al. 2012) from the CerealDB database (www.cerealsdb.uk.net) and (v) new SNP identified from the QTL region using Diversity Among Wheat geNome (DAWN), a wheat genomic platform (University of Adelaide) (Watson-Haigh et al. 2018). SNP were converted to KASP™ assays (He et al. 2014; Semagn et al. 2014). KASP™ primers were designed using the Kraken software from LGC genomics (www.lgcgroup.com) and assayed on parents and Excalibur/Kukri DH lines using a SNPLine (LGC genomics, www.lgcgroup.com/our-science/genomics-solutions/genotyping/kasp-genotyping-chemistry). We re-constructed the chromosome 1B genetic map by combining markers from the previous map with new markers using ICiMapping v 4 (Meng et al. 2015) with linkage criterion set to a LOD threshold > 3. Recombination frequencies were converted to cM using the Kosambi mapping function (Kosambi 1943), and the marker order was optimized using RECORD algorithm. Double crossovers were manually curated, and markers with high segregation distortion were discarded.

### Field experiments

A total of 32 field experiments were conducted at 10 locations on three continents over six seasons (Table S2). An experiment is defined as a location by year combination. Data on weather conditions for Australia were obtained from the Australian Bureau of Meteorology (www.bom.gov.au) for the nearest weather station to each experiment, and those for Mexico were obtained from the Centro de Investigaciones Agricolas del Noroeste (CIANO) meteorological station (Table 1). For the Australian sites of Booleroo Centre, Piednippie and Robinvale, the closest weather stations were 44, 20 and 80 km away, respectively. The remaining experiments had a weather station within 3 km. The meteorological data for Indian trials were collected from weather stations located on site.

**Table 1:** Growing season climatic data for the field trials

Country	Experiment	Rainfall (mm)	Number of days		Total daylight (h)	Day length (h)	
			< 10 °C	> 30 °C		Minimum	Maximum
Australia	Boo-06-rf	86	147	21	2018	9.9	14
	Boo-07-rf	191	158	16	2074	9.9	13.9
	Min-06-rf	68	116	10	1673	9.9	13.5
	Min-07-rf	74	120	12	1760	9.9	13.5
	Pie-07-rf	173	82	2	1585	9.9	12.9
	Ros-06-rf	131	154	12	1900	9.7	13.8
	Ros-07-rf	229	142	9	1873	9.7	13.7
	Rob-07-rf	99	135	7	1821	9.7	13.6
Mexico	Obr-07-rf	150	98	10	1531	10.3	12.7
	Obr-07-ir	500^a^	106	38	1924	10.3	13.4
India	His-11-ir	254	100	27	1650	10.3	12.8
	His-11-rf	74	100	27	1650	10.3	12.8
	His-12-ir	352	97	16	1611	10.3	12.7
	His-12-rf	172	97	16	1611	10.3	12.7
	Kan-10-ir	282	52	23	1499	10.5	12.5
	Kan-10-rf	102	52	23	1499	10.5	12.5
	Kan-11-ir	282	48	25	1561	10.5	12.5
	Kan-11-rf	102	48	25	1561	10.5	12.5
	Kan-12-ir	367	73	32	1592	10.5	12.5
	Kan-12-rf	187	73	32	1592	10.5	12.5
	Kar-10-ir	251	84	20	1530	10.2	12.5
	Kar-10-rf	71	84	20	1530	10.2	12.5
	Kar-11-ir	264	91	15	1477	10.2	12:06
	Kar-11-rf	84	91	15	1477	10.2	12.6
	Kar-12-ir	440	87	16	1550	10.2	12.8
	Kar-12-rf	260	87	16	1550	10.2	12.8
	Pun-10-ir	314	31	46	1476	11	11.7
	Pun-10-rf	134	31	46	1476	11	11.7
	Pun-11-ir	282	36	32	1535	11	11.8
	Pun-11-rf	102	36	32	1535	11	11.8
	Pun-12-ir	250	22	22	1547	11	11.5
	Pun-12-rf	70	22	22	1547	11	11.5

Experiments are named using the three first letters of the trial site (Booleroo, Minnipa, Piednippie, Roseworthy, Robinvale, Obregon, Hisar, Kanpur, Karnal, Pune), then the two last digits of the trial year followed by the irrigation conditions (rf: rainfed; ir: irrigated)

^a^An estimate of the water volume applied using flood irrigation

Eight experiments were conducted in South Australia in 2006 and 2007 at Booleroo Centre, Minnipa Agricultural Research Centre, Piednippie, Roseworthy Agricultural College, and Victoria in 2007 at Robinvale. The southern Australian field experiments included all 233 DH lines randomized using a nearest neighbour design with two replicates, with additional plots of the parental lines and control varieties which are well adapted to Southern Australia, including Axe, Carinya, Drysdale, Espada, Excalibur, Frame, Gladius, Kukri, Krichauff, RAC875, Stylet, Tincurrin, Westonia, Wyalkatchem and Yitpi. The Minnipa and Piednippie plots were 1.8 m wide and 7 m long with 8 rows. At the other southern Australian sites, the plots were 1.25 m wide and 5 m long, each with either 5 or 6 rows. Plots were reduced in length by herbicide application to 5 m in Minnipa and Piednippie and to 3.2 m at the other southern Australian sites, just prior to anthesis. Seeds were sown on a volume basis aiming for an average of 200 seeds m^−2^. The agronomic management regime followed local practices at each location.

In 2007, two experiments were conducted at CIANO, which is CIMMYT’s (International Maize and Wheat Improvement Centre) drought evaluation site in Ciudad de Obregon (north-western Mexico). The Mexican field experiments included all 233 DH lines randomized using an alpha lattice design with two replicates, the parental lines and one control variety that is well adapted to the growing area (Sokoll). The durum variety Atilla was also included as a filler wherever required. Two irrigation regimes created two contrasting environments. Drip irrigation simulated a southern Australian cyclical drought stress by applying three applications of approximately 50 mm each at sowing, and 28 and 40 days thereafter. Flood irrigation provided a high yielding, non-drought-stressed environment, with four applications applied to field capacity at sowing, and 48, 72 and 130 days thereafter. The plots were 0.8 m wide by 3.5 m long with 4 rows and 0.4 m wide by 2 m long with 2 rows in the drip and flood irrigated environments, respectively.

In India, data were collected from 18 field experiments in irrigated and rainfed environments over 3 years (2010–2011, 2011–2012 and 2012–2013) at four locations: Hisar, Kanpur, Karnal and Pune (Table S2), as described earlier (Gahlaut et al. 2017). Briefly, the experiments included 192 DH lines and were conducted in augmented block designs comprising 12 blocks with each block containing 19 DH lines and three control varieties that are adapted to Indian environments (NI5439, PBW175 and WH147). DH lines and controls in a block were evaluated in plots of 0.75 m^2^ with three rows of 1.5 m length and row-to-row distance of 25 cm. Irrigation is applied as detailed in Table 1.

The Excalibur/Kukri DH population was phenotyped for the following traits in all field trials: grain yield (Yld), thousand grain weight (TGW), days to anthesis (DTA), days to heading (DTH) and grain filling duration (GFD). DTH was determined from the date of sowing to the date at which 50–75% heads emerged from the flag leaf. DTA was calculated as the number of days between sowing and the date when 50–75% heads showed anthesis in a plot. The data on GFD were calculated as the difference in number of days between anthesis and physiological maturity. The plots were machine harvested and grain cleaned. The cleaned samples were weighed to calculate Yld expressed in kg/ha, and TGW was recorded in g by counting and weighing 1000 grains.

### Polytunnel NIL experiment

Five pairs of NIL (EK428_2, EK428_8, EK570, EK405 and EK664) and the two parental lines (Excalibur and Kukri) were grown in the field using a rainout shelter (polyurethane) and drip irrigation to evaluate plant performance under southern Australian conditions of 2016 (Urrbrae, South Australia). The air temperature and soil water potential are recorded and shown in Fig. S1. The material was planted on August 5th, which is later than farmers practice (usually around May) to ensure a terminal drought and heat stress after heading. Forty-eight plants of each NIL were grown in mini-plots of 6 rows in a plot of 0.6 m by 0.8 m with a space of 10 cm between plants within a row and 10 cm between rows. The experiment was arranged in a fully randomized complete block design with two replications. Drought treatment was induced by withholding irrigation at early booting stage (41 score on the Zadoks’ scale) (Zadoks et al. 1974). Soil water potential was measured using six gypsum blocks (MWS model, Hunter Industries, Australia) that were installed at three positions (3 m apart) of the trial and in two different soil depths (15 cm and 40 cm). Temperature and humidity data were recorded using a mobile logger (KG100 model, Adelaide, Australia) positioned at three equal distance (3 m apart) in the field.

NIL were phenotyped for the following traits: Yld, TGW, DTH, DTA, GFD, days to maturity (DTM), normalized differences in vegetative index (NDVI), above ground biomass (BM), harvest index (HI), fertile tillers/plot (FT), screenings (Scr) and total grain number/plot (TGN). DTH was recorded as the number of days from planting to 50% plants reaching heading, DTA was recorded as days to anthesis, and DTM was recorded as days to physiological maturity. NDVI was recorded at vegetative stage (tillering to booting stage) by capturing the reflectance spectra of the canopy with a portable spectroradiometer, GreenSeeker™ (NTech Industries Ins, Ukiah, California, USA) as described by Gutiérrez-Rodríguez et al. (2004). The GreenSeeker™ sensor was held at 0.4–0.6 m above the canopy to scan 0.48 cm^2^ mini-plots. A thin metal string with a pointer at the end was hung at the tip of the GreenSeeker to keep it at a constant distance above the canopy during scanning. NDVI data were measured from 10:00 am to 1:00 pm. Above ground biomass (BM) was harvested at maturity and weighed (g). Plant height (PH) was measured in metres on ten plants per mini-plot from the ground to the top of the spike excluding awns. One spike, each from 10 random plants were sampled from the middle of each mini-plot and threshed to measure grains/spike (GS), grains/spikelet (GSp) and spikelets per spike. A 2.0-mm sieve (Graintech scientific, Queensland, Australia) was used to screen the grains and measure screenings (Scr, %). Scr is the ratio of the weight of the grains passing through the screen to the weight of total grains per plot and multiplied by 100. TGW was measured in grams by weighing 500 randomly sampled seeds after screening. Grain number per plot (GNp) was counted using a seed counter (Pfueffer GmBH, Germany) after seed screening. HI was calculated as the ratio between Yld and BM.

### Multi-environment QTL analysis

We used a mixed model methodology for multi-environment trials (MET) to investigate QTL main effects as well as *Q* * *E* interaction (Malosetti et al. 2013; Bonneau et al. 2013). These analyses were performed using ASReml-R package that estimates variance components under a linear mixed model by residual maximum likelihood (Butler et al. 2009, http://www.vsn.co.uk), in the R Statistical Computing Environment (R Core Team 2016).

Environments refer to a unique combination of geographic location and year. For the analysis of each phenotypic trait in an environment, the following model was defined:

1 $$\begin{array}{*{20}c} {y = X\tau + Zu + \varvec{Z}_{\varvec{g}} g + e\# } \\ \end{array}$$

where $\varvec{y}$ represents the vector of trait values. The fixed effects components $\varvec{\tau}$ differentiated the DH lines from the control lines such as parents and cultivars, effects pertaining to agricultural practices (such as the side of seeding and the direction of scoring, if available) and possible linear trends across rows or columns at each environment. Random effects $\varvec{u}$ modelled random non-genetic effects pertaining to spatial trends (Gilmour et al. 1997) in each environment.

Residual variance in each environment was assumed to have a distribution $e \sim N\left( {0, \sigma^{2} R} \right)$ where $R$ is a correlation structure for a separable autoregressive process of first order (AR1 × AR1 process) across rows and columns in each of the field trials.

The random effects $\varvec{g}$ model the effect of genotypes at each trial location and were assumed to be distributed as $N\left( {0, \sigma_{g}^{2} I_{g} } \right)$ where $\sigma_{g}^{2}$ is the genetic variance and $I_{g}$ are identity matrices. This term accounted for the combined overall genetic basis of a phenotypic trait.

Initially, the model with diagonal variance structure for the genotype effects was fitted (i.e. assuming un-correlated genetic effects in different field trials) and used to evaluate the amount of genetic variance within each field trial. The data for field trials with very low genetic variance (< 1% of the residual variance) were removed from further analysis; such trials mainly included trials conducted in India (8/18 trials for yield, 5/18 trials for TGW and 2/18 trials for GFD).

In the final MET model, variance–covariance matrix for the *G* * *E* interaction was modelled with factor analytic (FA) structure that approximates the unstructured matrix (Smith et al. 2001), with initial parameter values provided by the diagonal model, and each subsequent FA model using the previous FA model’s values until overall percentage between environment genetic variance exceeded 80% (Smith et al. 2015). The final FA mixed model was used to assess genetic correlations across field trials and to calculate a generalized heritability $h_{g}^{2}$ according to the formula by Cullis et al. (2006) and Oakey et al. (2006):

$$h_{g}^{2} = 1 - \frac{\text{PEV}}{{2\sigma_{g}^{2} }}$$

where PEV is the average pairwise prediction error variance of the BLUPs and $\sigma_{g}^{2}$ is the genetic variance.

A simple genome-wide interval mapping was conducted, where a genetic predictor, represented by an estimated genetic marker interval was fitted—one at the time—in the fixed components of FA mixed model; the model (1) was extended as:

2 $$\begin{array}{*{20}c} {y = X\tau + X\varvec{E}_{\varvec{j}} + X\varvec{\alpha}_{\varvec{m}} + X\varvec{\alpha}_{{{\mathbf{QEI}}}} + Zu + \varvec{Z}_{\varvec{g}} g + e\# } \\ \end{array}$$

where $\varvec{XE}_{\varvec{j}}$ is the fixed effect of the *j* environment, $\varvec{X\alpha }_{\varvec{m}}$ is the fixed main effect of the genetic marker predictor and $\varvec{X\alpha }_{{{\mathbf{QEI}}}}$ is the fixed effect for the interaction between the genetic predictor and the environment *j*, or *Q* * *E* interaction.

The genetic marker intervals used as genetic predictors represented the expectation of the genotype given the imputed (as in Martinez and Curnow 1994) flanking markers and were calculated based on recombination fractions (as detailed in Verbyla et al. 2007) using the functionalities of R/wgaim package (Taylor and Verbyla 2011). The genetic marker intervals were coded as − 1 if the genotypic value of a line was homozygous for parent Kukri or 1 if homozygous for parent Excalibur. Consequently, a negative value of the mixed model coefficient for the effect of genetic predictor indicated that the allele coming from parent Kukri increases the trait, while a positive value indicated that the allele coming from the parent Excalibur increases the trait.

The significance of the genetic predictor was tested with Wald test for the null hypothesis of the effect of the genetic predictor being zero across all environments. For interval mapping, a threshold of *p* = 0.01 for *p* value of the Wald test and the set of genetic predictors representing genetic positions with postulated QTLs were selected if the *p* value for the marker effect $\varvec{X\alpha }_{\varvec{m}}$ and/or the QEI effect $\varvec{X\alpha }_{{{\mathbf{QEI}}}}$ were below the threshold. The threshold of 0.01 was chosen due to the exploratory nature of study.

The above analysis was first conducted for DTH to detect QTL for heading date. We then used the markers *Vrn1A* on chromosome 5A, 1228158.44AG and 1127751.6TC on chromosome 7A (which are linked to the QTL *QDth.aww*-*5A*, *QDth.aww*-*7A.1* and *QDth.aww*-*7A.2*) as DTH-related covariates and fitted them as additional fixed effects of the model (2) in the analyses of yield, TGW and GFD. To avoid collinearity with the tested marker, covariates that were on the chromosome being evaluated were excluded (Malosetti et al. 2013).

### Sequence annotation of the yield QTL interval on chromosome 1B

The sequences of the *QYld.aww*-*1B.2* flanking markers *adw1218477* and *BS00022342* were aligned to the reference sequence of Chinese Spring (IWGSC RefSeq v.1.0) by BLASTN through the URGI portal (https://wheat-urgi.versailles.inra.fr/) to identify the physical position of the QTL. The protein sequences of the genes in the QTL interval were obtained from Emsembl Plants (http://plants.ensembl.org/index.html) and used for homology search (BLASTP) in rice (*Oryza sativa*) and Brachypodium (*B. distachyon*) using Phytozome V12.1, the Plant Comparative Genomics portal, Department of Energy’s Joint Genome Institute (https://phytozome.jgi.doe.gov/pz/portal.html). Descriptions for the wheat predicted genes were obtained from DAWN (Watson-Haigh et al. 2018).

## Results

### Phenotypic performance of DH mapping population

**Fig. 1:**
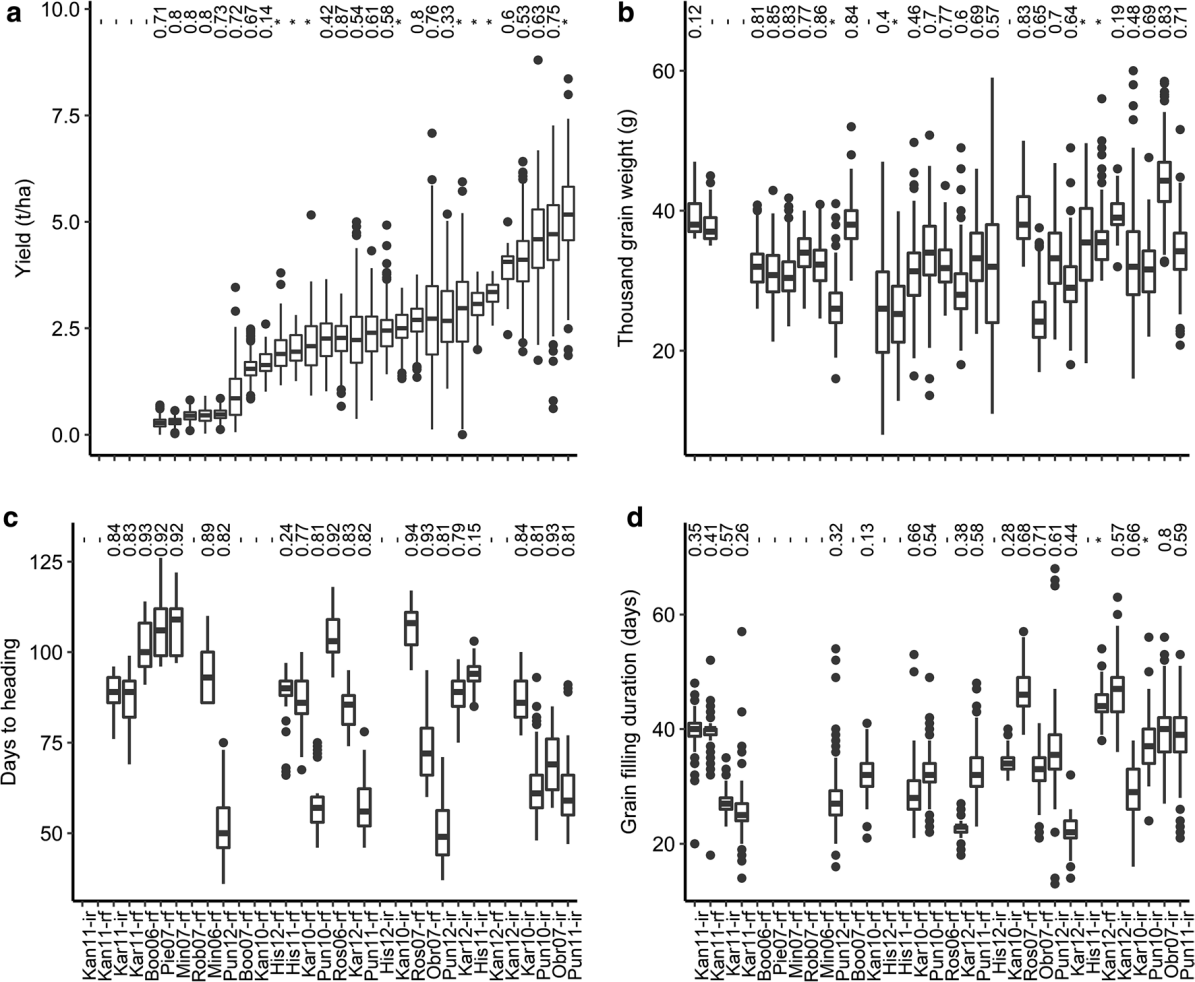
Variation for grain yield (**a**), thousand grain weight (**b**), days to heading (**c**) and grain filling duration (**d**) among the Excalibur × Kukri DH lines in field trials. In the boxplot, the solid horizontal line indicates median value, the box indicates the second and the third quartiles, whiskers indicate 1.5 ± interquartile range, dots indicate outliers. Heritability estimates in each environment derived from the FA models are presented on top of the figure. Note that the trait was not measured in some of the environments (indicated by hyphen) or genetic variance was much lower than residual variance as estimated from the associated FA models (indicated by asterisk, see also statistical analysis). *Boo* Booleroo 2006 and 2007, *Obr* Obregon 2007, *Min* Minnipa 2006 and 2007, *Ros* Roseworthy 2006 and 2007, *His* Hisar 2011 and 2012, *Kan* Kanpur 2010, 2011 and 2012, *Pun* Pune 2010, 2011 and 2012, *rf* rainfed, *ir* irrigated trial

The broad range of environmental conditions across 32 experiments provided varying water conditions ranging from 68 mm to 500 mm of water supply (Table 1). The trials with the highest number of hot days (> 30 °C) were in Obregon, Mexico, and in Hisar, Kanpur and Pune, India. The average grain yields ranged from 0.3 to 6.0 t/ha with a mean grain yield of 2.5 t/ha (Fig. 1). Six experiments in the Australian rainfed environments of Booleroo and Minnipa in 2006, Minnipa, Piednippie and Robinvale in 2007, and also in the rainfed Indian trial at Pune in 2012 showed very low average yield (< 2 t/ha); these trials experienced severe drought having received only 68–173 mm rainfall. The trials in Obregon 2007, Kanpur 2012, Karnal 2010 and Pune 2010 and 2011 showed higher than average yield and were all irrigated. These experiments received 251–500 mm of water in the cropping cycle, and despite experiencing the longest period of hot days (with 32–46 days > 30 °C), these trials yielded > 4 t/ha with some lines yielding > 7 t/ha. Heritability for yield ranged from 0.14 to 0.87; the heritability was higher in Australian and Mexican trials overall and wasn’t related to the average yield at the trial (Fig. 1).

For DTH, the genetic correlations between experiments showed two distinct groups of environments, one composed of Australian and Mexican trials and the other comprising the Indian trials (minus two trials in Hisar) (Fig. S2). This probably reflects the differences in phenology requirement across the three continents. The grouping was very similar for TGW but differs slightly for grain yield. Australian trials correlated well with each other for grain yield (Fig. S2). Indian trials formed a second group with similar grain yield ranking and were negatively correlated to a third group of two trials in India (Pune and Kanpur 2012) and an irrigated trial in Mexico. The trials under hot and humid conditions giving high average yield including Obregon 2007, Kanpur 2012, Karnal 2010 and Pune 2010 and 2011 did not correlate with each other; the experiments rather clustered in geographical locations rather than with level of water supply and number of hot days (Fig. S2).

### QTL for yield-related traits in the DH population

**Fig. 2:**
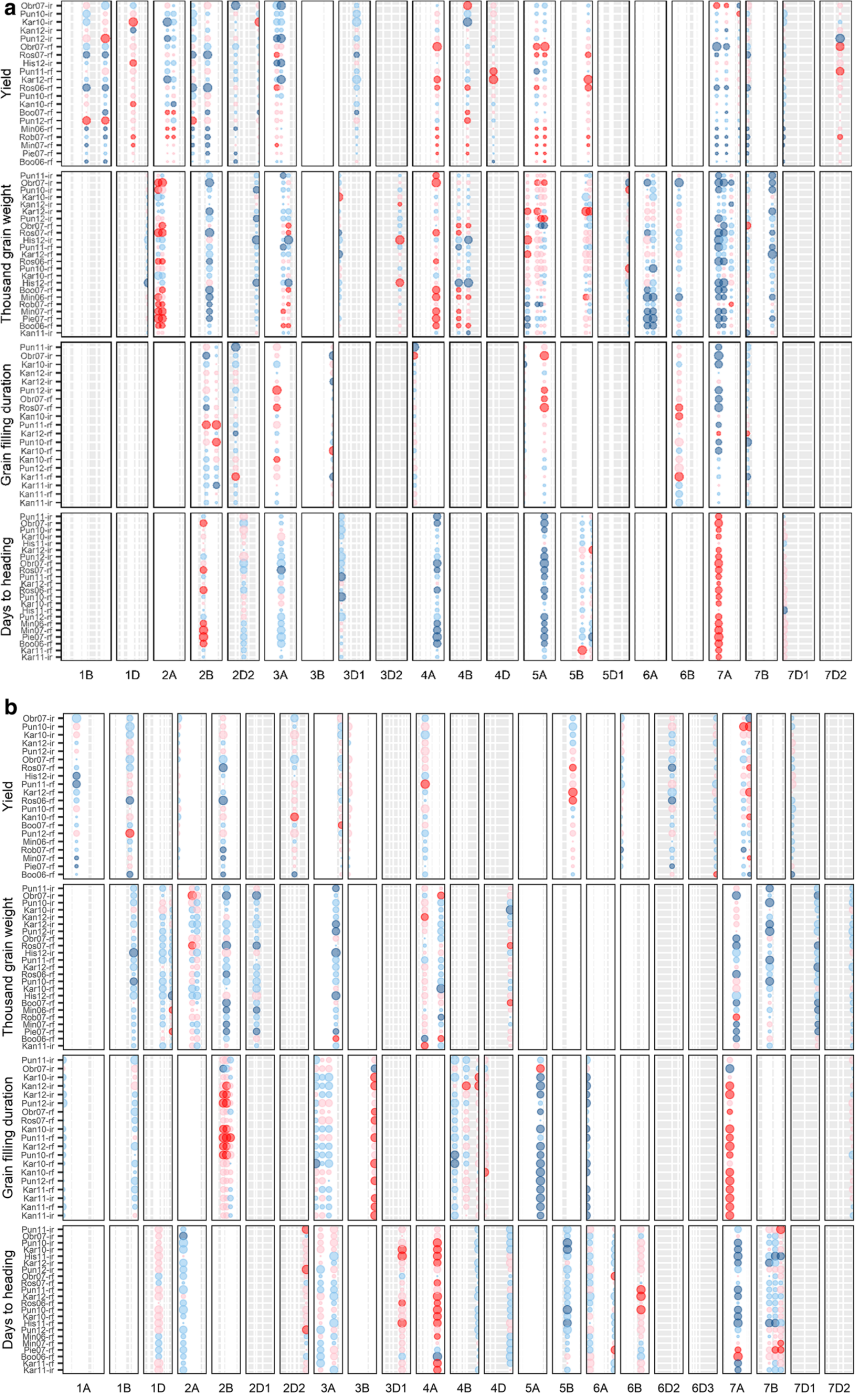
QTL for grain yield, thousand grain weight, grain filling duration and days to heading in the Excalibur/Kukri DH population with significant **a** Q * E effect or **b** main effect in multiple environments. Blue colour indicates that Excalibur allele increases the trait value at a QTL, pink indicates Kukri allele increases the trait value. The area of the circle is proportional to the magnitude of the estimated QTL effect on the trait, with the effects significantly different from zero indicated by stronger shade of either blue or pink. The trials on the *y*-axis are sorted according to the mean yield per trial. The highest mean yield per trial was recorded in Pun11-ir followed by Obr07-ir; however, the genetic variance for yield for yield in Pun11-ir was very low, and therefore this trial was excluded from the genome-wide QTL analyses of yield. The same was true for His11-ir, His11-rf, His12-rf, Kan10-ir, Kan12_rf, Kar10_rf, Kar12_ir and these trials do not appear on the y-axis of the panel for yield. Additionally, yield was not phenotyped in trials Kan11-ir, Kan11-rf, Kar11-ir and Kar11-rf; whenever present for other traits, these four trials are shown first on the *y*-axis. Some chromosomes are represented by multiple linkage groups (due to gaps in coverage), and their names reflect their relative position (e.g. 3D1 and 3D2) on wheat pseudomolecule (colour figure online)

The multi-environment analysis with modelling for phenology effect enabled the identification of yield QTL that are independent of phenology loci known to segregate in the Excalibur/Kukri population (Hill et al. 2013, 2015). A total of 128 QTL were identified for four traits analysed across 32 experiments (Fig. 2); the QTL were spread over the whole genome. Of these 128 QTL, 24 QTL for yield, 27 for TGW (Table 2), 10 for DTH and 11 for GFD (Table S3) showed significant *Q* * *E* interactions. Overall, we found more QTL with *Q* * *E* effects (Fig. 2a) than QTL with significant main effects for yield and TGW across environments (Fig. 2b). The yield QTL with the highest QTL effects were observed on chromosomes 4A, 5B and 7A with a yield increase of up to 1324 kg/ha (for *QYld.aww*-*7A.1*); these strong QTL are environment specific, with strong *Q* * *E* (Fig. 2a). QTL for yield, TGW and DTH overlap on chromosome 4A, indicating that this locus might be controlled by phenology, by contrast with the loci on 5B and 7A where QTL co-located for yield and TGW but not DTH. Looking at the data for average yield under three environments (hot and irrigated trials; rainfed trials with mid-range yield; trials under severe drought), no QTL specific for abiotic stress were found.

**Table 2:** QTL for grain yield and thousand grain weight in Excalibur/Kukri DH population

Chr	QTL	Flanking markers	QTL peak	Term	*P* value	QTL effect
Grain yield						kg/ha
1A	*QYld.aww*-*1A*	X3021844–gwm0558	45.2–46.8	Main effect	< 0.001	15.3
1B	*QYld.aww*-*1B.1*	X1134677–X1054439	60.2–66.1	*Q* * *E*	< 0.01	− 274 to 173
1B	*QYld.aww*-*1B.2*	X2263055–X1061145	84.2–84.9	Main effect	< 0.01	14.5
1B	*QYld.aww*-*1B.3*	Xwmc0830–Xpsp3100	105.3–110.5	*Q* * *E*	< 0.01	− 240 to 127
1D	*QYld.aww*-*1D*	X2243386–1126683	70.3–84.1	*Q* * *E*	< 0.01	− 305 to − 113
2A	*QYld.aww*-*2A.1*	X1128252–X985175	0–2.4	Main effect	< 0.01	− 11.1
2A	*QYld.aww*-*2A.2*	X1051900–X1072243	71.8–72.5	*Q* * *E*	< 0.001	− 79 to 395
2A	*QYld.aww*-*2A.3*	Xwmc0602–X1131447	103.6–105.7	*Q* * *E*	< 0.01	− 61 to 121
2B	*QYld.aww*-*2B.1*	X3064619–X100005743	9.1–16.4	*Q* * *E*	< 0.0001	− 175 to 200
2B	*QYld.aww*-*2B.2*	X1075921–X3021359	52.2–52.9	Main effect	< 0.01	11.3
2B	*QYld.aww*-*2B.3*	X1102573–X1010970	70.4–71	*Q* * *E*	< 0.0001	37–238
2D	*QYld.aww*-*2D.1*	X2242328–X991014	19.4–28.2	*Q* * *E*	< 0.001	38–323
2D	*QYld.aww*-*2D.2*	wPt3728–wPt0638	38.9–40	Main effect	< 0.0001	25.3
2D	*QYld.aww*-*2D.3*	wPt1301–X100006360	76.2–76.3	*Q* * *E*	< 0.01	− 335 to 62
3A	*QYld.aww*-*3A.1*	X2254081–X1086149	63.3–64.3	*Q* * *E*	< 0.001	− 126 to 194
3A	*QYld.aww*-*3A.2*	X1132581–X1014677	85.8–86.5	*Q* * *E*	< 0.01	225–315
3A	*QYld.aww*-*3A.3*	X1026080–991604	157.5–158.8	Main effect	< 0.01	− 16.1
3B	*QYld.aww*-*3B*	X989831–X1207987	2–2.6	Main effect	< 0.01	− 13.1
3DSL	*QYld.aww*-*3DSL*	1056891–wPt6262	48.1–59	*Q* * *E*	< 0.01	61–104
4A	*QYld.aww*-*4A.1*	X979934–X1042486	53.5–56	Main effect	< 0.0001	19.9
4A	*QYld.aww*-*4A.2*	X3064552–X1125529	130–142.7	*Q* * *E*	< 0.0001	− 580 to − 34
4B	*QYld.aww*-*4B*	gwm0495–ksm0154	43.4–45.5	*Q* * *E*	< 0.0001	− 449 to 507
4D	*QYld.aww*-*4D*	X2256312–X1022538	8–14.7	*Q* * *E*	< 0.01	− 471 to 24
5A	*QYld.aww*-*5A.1*	X1097973–X978326	77.7–79.6	*Q* * *E*	< 0.01	− 338 to 229
5A	*QYld.aww*-*5A.2*	Vrn1A–X1264710	125.5–126.2	*Q* * *E*	< 0.0001	− 521 to − 22
5B	*QYld.aww*-*5B.1*	X1032121–X3026027	115.1–115.9	Main effect	< 0.0001	29.7
5B	*QYld.aww*-*5B.2*	X3020443–X2261812	139.8–143.1	*Q* * *E*	< 0.0001	− 561 to − 36
6B	*QYld.aww*-*6B*	X996294–X1220113	0–1.9	Main effect	< 0.01	15.2
6DLbot	*QYld.aww*-*6DLbot*	X2244522–X990024	14.3–17.2	Main effect	< 0.0001	− 17.5
6DLtop	*QYld.aww*-*6DLtop*	X3028493–X995315	3.7–5.2	Main effect	< 0.01	− 17.5
7A	*QYld.aww*-*7A.1*	barc1055–1246868	45.1–56.3	*Q* * *E*	< 0.0001	− 517 to 1324
7A	*QYld.aww*-*7A.2*	X1116135–9K6004	99.4–100.7	*Q* * *E*	< 0.0001	− 457 to 357
7A	*QYld.aww*-*7A.3*	X2262955–wmc633	129.2–137.6	Main effect	< 0.001	25.3
7A	*QYld.aww*-*7A.4*	X2341144–X987692	171.5–174.5	*Q* * *E*	< 0.0001	− 814
7B	*QYld.aww*-*7B*	X987292–X1083752	6.8–29.2	*Q* * *E*	< 0.01	36–163
7DS	*QYld.aww*-*7DS.1*	WPT2551–WPT0366	0–1.1	*Q* * *E*	< 0.001	33–68
7DS	*QYld.aww*-*7DS.2*	WPT5049–gdm0088	1.8–25.4	Main effect	< 0.01	21.8
7DSL	*QYld.aww*-*7DSL*	X3022993–stm0789tcacD	13.6–21.1	*Q* * *E*	< 0.01	− 218 to 235
*Thousand grain weight*						*g*
1B	*QTgw.aww*-*1B*	X1005607–X2263979	100–100.2	Main effect	< 0.001	0.4
1D	*QTgw.aww*-*1D.1*	wPt1799–1129001	88.7–102.5	Main effect	< 0.001	0.4
1D	*QTgw.aww*-*1D.2*	X988357–X1108800	133.9–135.3	*Q* * *E*	< 0.001	− 1 to 3
2A	*QTgw.aww*-*2A.1*	X1133336–X1056356	24.9–30.5	*Q* * *E*	< 0.001	− 2 to 2
2A	*QTgw.aww*-*2A.2*	X2277859–X2256686	45.9–50.1	*Q* * *E*	< 0.001	− 2 to − 1
2A	*QTgw.aww*-*2A.3*	X1132500–X1094923	83.4–84.8	Main effect	< 0.01	− 0.3
2A	*QTgw.aww*-*2A.4*	X1061775–X1038091	110.2–111.5	Main effect	< 0.01	− 0.3
2B	*QTgw.aww*-*2B.1*	X1022997–X1082017	67.2–67.8	Main effect	< 0.0001	0.6
2B	*QTgw.aww*-*2B.2*	X2266150–X990132	78.2–78.8	*Q* * *E*	< 0.001	1–2
2D	*QTgw.aww*-*2D*	X1086964–X1046316	69.7–70.5	*Q* * *E*	< 0.001	1–4
2DS	*QTgw.aww*-*2DS*	X1162627–X1128261	2.6–4.3	Main effect	< 0.0001	− 0.4
3A	*QTgw.aww*-*3A.1*	X1130383–wPt9562	96.5–97.2	*Q* * *E*	< 0.001	− 1 to 2
3A	*QTgw.aww*-*3A.2*	cfa2170–X1205035	123.2–123.9	*Q* * *E*	< 0.001	− 1 to 4
3DL	*QTgw.aww*-*3DL*	X1234793–X3026282	1.3–1.9	*Q* * *E*	< 0.0001	− 5 to 1
3DSL	*QTgw.aww*-*3DSL*	X1109431–wPt2313	0–7.4	*Q* * *E*	< 0.001	− 3 to 2
4A	*QTgw.aww*-*4A.1*	X3020781–X1072050	51.3–51.5	Main effect	< 0.01	0.3
4A	*QTgw.aww*-*4A.2*	X1242399–X3064552	124.4–130	*Q* * *E*	< 0.0001	− 3 to − 1
4B	*QTgw.aww*-*4B.1*	wmc0047–wmc0349	22.1–23.5	*Q* * *E*	< 0.01	− 2 to 5
4B	*QTgw.aww*-*4B.2*	ksm0154–DuPw0036	45.5–72.7	*Q* * *E*	< 0.001	− 2 to 6
4D	*QTgw.aww*-*4D*	X1004846–X1161775	35.2–37.8	Main effect	< 0.0001	− 0.4
5A	*QTgw.aww*-*5A.1*	wPt9887–X1094095	23.3–35.8	*Q* * *E*	< 0.01	− 3 to 1
5A	*QTgw.aww*-*5A.2*	wPt8226–X1070217	81.8–83.1	*Q* * *E*	< 0.01	− 2 to 1
5A	*QTgw.aww*-*5A.3*	X1061673–X1093737	103.2–111	*Q* * *E*	< 0.001	− 2 to 1
5A	*QTgw.aww*-*5A.4*	X1135154–Vrn1A	123.6–125.5	*Q* * *E*	< 0.01	− 2 to 2
5B	*QTgw.aww*-*5B.1*	X3023130–X1217242	128.2–130.2	*Q* * *E*	< 0.01	− 2 to − 1
5B	*QTgw.aww*-*5B.2*	X1227599–X1240475	146.9–161.5	*Q* * *E*	< 0.01	− 2 to 1
5DSL	*QTgw.aww*-*5DSL*	cfd0019–5D–X1102031	94–96.2	*Q* * *E*	< 0.01	− 2 to 2
6A	*QTgw.aww*-*6A.1*	X1081530–GW2	40.1–43.3	*Q* * *E*	< 0.001	1–2
6A	*QTgw.aww*-*6A.2*	stm0544acag–X1325639	58.2–66.1	*Q* * *E*	< 0.001	1–2
6B	*QTgw.aww*-*6B*	X1075699–wPt5408	39.1–39.9	*Q* * *E*	< 0.01	1–2
7A	*QTgw.aww*-*7A.1*	1246868–X1228158	56.3–56.9	*Q* * *E*	< 0.0001	1–4
7A	*QTgw.aww*-*7A.2*	X3064804–barc0070	85.1–85.7	*Q* * *E*	< 0.0001	1–3
7A	*QTgw.aww*-*7A.3*	9K6680–X2262955	126.6–129.2	*Q* * *E*	< 0.001	− 1 to 1
7B	*QTgw.aww*-*7B.1*	X987292–X1083752	6.8–29.2	*Q* * *E*	< 0.01	− 2 to 1
7B	*QTgw.aww*-*7B.2*	X981613–X2279417	59.6–64.8	Main effect	< 0.01	− 0.3
7B	*QTgw.aww*-*7B.2*	BSm3603_7B–wPt6156	109.9–110.6	*Q* * *E*	< 0.001	1–3
7DS	*QTgw.aww*-*7DS*	X1103334–barc0092	50.2–52.3	Main effect	< 0.01	− 0.4
7DSL	*QTgw.aww*-*7DSL*	stm0789tcacD–wPt0789	21.1–29.1	Main effect	< 0.01	− 0.2

*P* value is derived from the Wald test for the effect indicated by Term (QTL main effect or QTL by environment interaction *Q* * *E*). QTL effect is the estimate of the effect indicated by Term, with a range of the estimates per environment that were significantly different from zero shown for *Q* * *E* effect (confidence interval of the estimate calculated as CI = estimate ± 1.96 * SE). Positive number shows allelic effect from Excalibur, negative number from Kukri

**Fig. 3:**
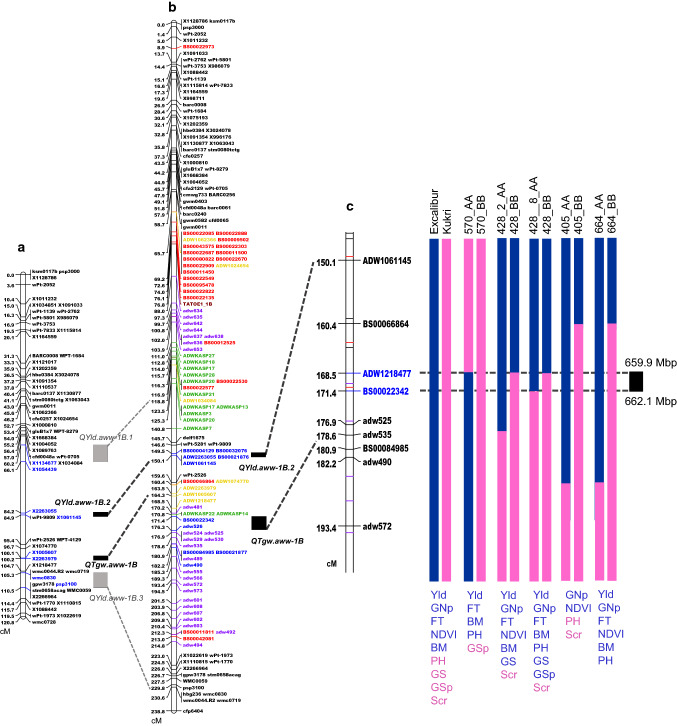
Fine mapping of *QYld.aww*-*1B.2* in wheat. **a** Low-resolution genetic map of chromosome 1B in Excalibur/Kukri DH population with QTL position from Table 2 (blue markers show markers flanking QTL peak). **b** High-resolution DH genetic map of chromosome 1B showing SSR-DArT and GBS markers (black), BS markers (red), GBS converted to KASP markers (yellow), KASP markers from 90 K Wheat Illumina Infinium iSelect (Comai et al. 2004) and new KASP markers (purple). **c** Genotype of five NIL pairs (blue: Excalibur allele; pink: Kukri allele) aligned to Excalibur/Kukri DH genetic map and showing the QTL interval on Chinese Spring RefSeq v.1.0 reference sequence (black box). Traits that are significantly different (also reported in Fig. 4) within a NIL pair are shown in blue when the positive allele comes from Excalibur and pink when it comes from Kukri. *Yld* yield, *GNp* grains number per plot, *FT* fertile tillers, *BM* biomass, *NDVI* normalized difference vegetative index, *GS* grains/spike, *GSp* grains/spikelet, *SpS* spikelet/spike, *PH* plant height, *Scr* screenings (colour figure online)

Fourteen yield QTL and 11 TGW QTL had significant main effects across environments (Table 2; Fig. 2b). These QTL with stable effects across trials have low effects on yield, with allelic effects ranged from 11.1 to 29.7 kg/ha (Table 2). The present study was also aimed to fine map a stable QTL controlling yield per se, i.e., a yield QTL with low *Q* * *E* effect and independent of phenology. Among 14 yield QTL, each with a significant main effect (Table 2), we excluded yield QTL that co-located with known loci such as plant height (*Rht*) or phenology (*Ppd* or *Vrn*) or with a QTL for DTH or GFD (Table S3). QTL for yield and TGW coincided with QTL for DTH on chromosomes 4A, 4D, 7A and 7B and so are likely to be phenology-dependent. We found two regions where QTL for yield and TGW overlap without DTH or GFD effects, on chromosomes 1B and 4A. We focused on a region of chromosome 1B where two QTL with non-significant *Q* * *E* interactions were found for yield and TGW with Excalibur as the source of the favourable alleles: *QYld.aww*-*1B.2* with a peak at 84.2–84.9 cM and *QTgw.aww*-*1B* at 100.1–100.2 cM (Table 2; Fig. 3a).

### Fine mapping of yield-related QTL on chromosome 1B

One hundred forty-two markers were added to chromosome 1B genetic map, resulting in a final map, which was 238.8 cM long with an average distance between two adjacent markers being 1.68 cM (Fig. 3b). GBS markers with the label X in Fig. 3a were converted to KASP assays and labelled ADW in Fig. 3b and c. Re-analysis of the yield data from 20 experiments with the new high-resolution 1B genetic map resulted in the identification of two main effect QTL: one flanked by the markers *BS00004129* (149.5 cM) and *ADW1061145* (150.1 cM) in Fig. S3a which corresponds to *QYld.aww*-*1B.2* in Table 2 and Fig. 3b, and a minor QTL peak at 180.9–182.2 cM (Fig. S3a). *QTgw.aww*-*1B* was mapped again with a peak flanked by markers at 171.4 and 176.9 cM (Fig. 3b and Fig. S3b). We used eight markers located in the region between 150.1 cM and 193.4 cM, which included *QYld.aww*-*1B.2* and *QTgw.aww*-*1B*, for NIL development (Fig. 3c).

In order to validate the QTL effects, we developed five pairs of NIL (EK570, EK428_2, EK428_8, EK405 and EK664) which segregated for markers within the 150.1–193.4 cM interval (Fig. 3c). Genotyping of the NIL with 22 KASP markers spread over the genome showed that the genetic background of EK570 NIL was fixed with half of the alleles coming from Excalibur and half from Kukri (Table S1). EK428 NIL contains the same genetic background, overall similar to Excalibur and showed different recombination points in a region of 10.1 cM between *ADW1218477* and *adw535* on chromosome 1B. EK405 and EK 664 NIL showed the same recombination point on 1B between *BS00066864* and *BS00021877*, but differed for the other chromosomes, with EK405 containing mostly Excalibur alleles while EK664 was similar to Kukri (Table S1). In addition, EK405 NIL differed for another locus (BS00072058) on chromosome 2D (Table S1).

The five pairs of NIL were phenotyped in semi-controlled field conditions under a single combined drought and heat treatment. The NIL were exposed to a severe drought of < − 0.6 MPa recorded at the top 10 cm soil profile starting from heading stage with a maximum temperature between 32.5 and 42.3 °C throughout flowering and grain filling (Fig. S1). The average yield across the whole trial (including NIL and parents) was 1.4 t/ha, which is close to the average yield in Australia (1.7 t/ha). No significant spatial effects could be detected across the trial, so no correction was applied for row and column positions of the lines.

**Fig. 4:**
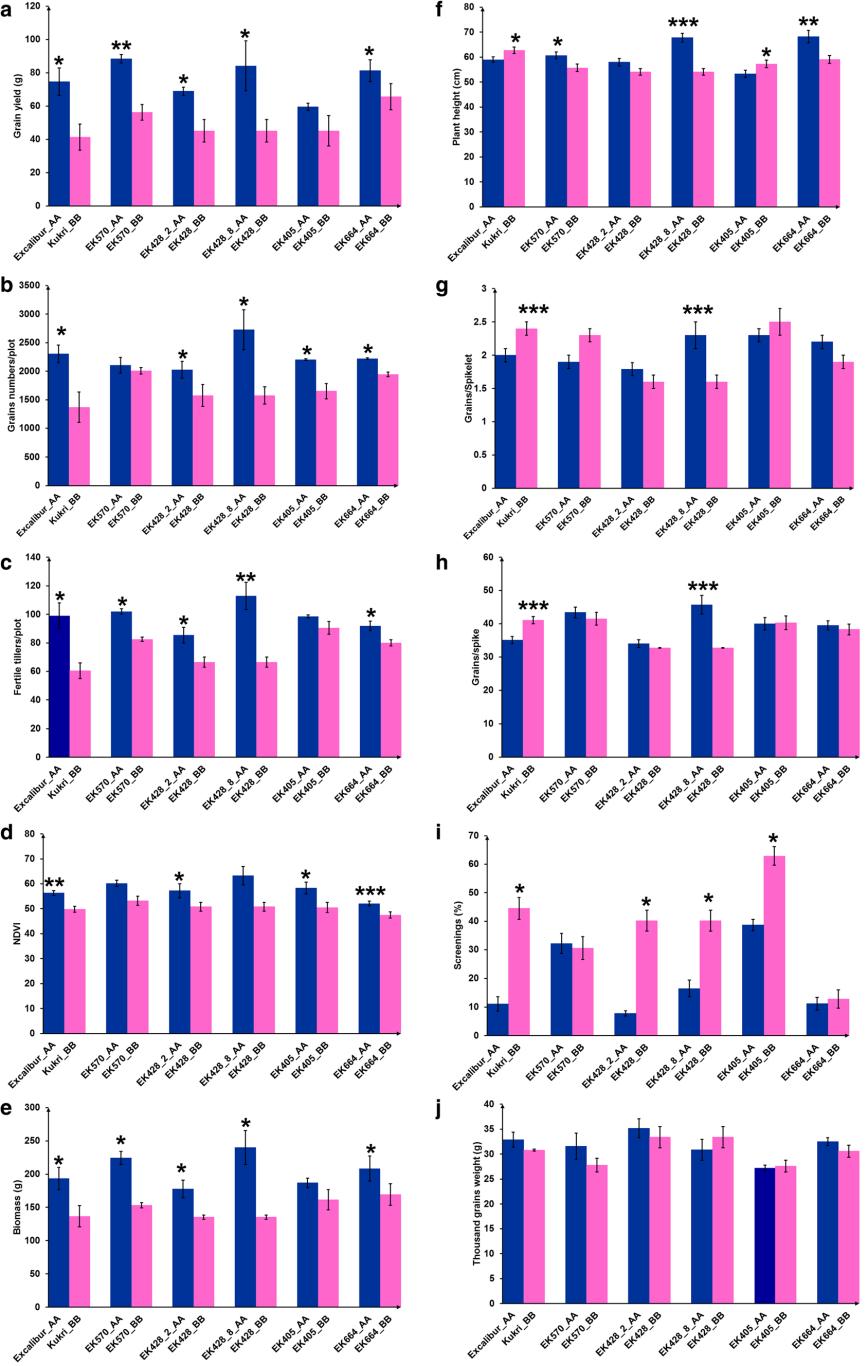
Phenotyping of five pairs of NIL segregating for *QYld.aww*-*1B.2* and grown under severe drought and hot conditions (Urrbrae, SA, 2016). Grain yield (**a**), total grains number/plot (**b**), fertile tillers (**c**), NDVI (**d**), biomass (**e**), plant height (**f**), grains/spikelet (**g**), grains/spike (**h**), screenings (**i**) and TGW (**j**). Differences between the mean of each NIL within a pair was evaluated at specific marker in the QTL region using *t* test. *, **, *** means significant difference at *p* < 0.05, 0.01 and 0.001 level, respectively

Excalibur showed significantly higher values than Kukri for grain yield, grain number/plot, number of fertile tillers/plot, NDVI and biomass, as also reported by Izanloo et al. (2008) (Fig. 4a–e). Kukri plants were taller with longer spikes, more grains per spike, but smaller grain size (higher screenings) than Excalibur (Fig. 4f–i). Statistical analysis of NIL and parental lines showed highly significant, intermediate to strong positive correlations among grain yield, biomass, grains number/plot, NDVI, fertile tillers, TGW and plant height (Table S4). Screenings (Scr) were negatively correlated to grain yield, grain number/plot and TGW.

In at least three NIL pairs, the Excalibur (AA) allele contributed the positive effect for the following traits: grain yield, grains number/plot, fertile tillers/plot, NDVI and biomass (Fig. 4a–e). Maturity traits (DTH, DTA and DTM) and TGW were not significantly different within NIL pairs confirming that *QYld.aww*-*1B.2* is independent of phenology and suggesting that *QTgw.aww*-*1B* had no effect on TGW in the developed NIL. Most NIL pairs showed significant differences in yield or yield components between AA and BB alleles (Fig. 4a–i). Four NIL pairs (EK570, EK428_2, EK428_8 and EK664) exhibited significant differences in grain yield, thus validating the effect of the Excalibur allele at *QYld.aww*-*1B.2* under severe drought and heat stress (Fig. 4a–i). Although the NIL pair EK405 did not show significant differences for grain yield (Fig. 4a), it follows the same trend as the other four NIL with Excalibur allele increasing grains number/plot and NDVI, and decreasing screenings (Fig. 4g–i).

Grain yield (Fig. 4a) significantly co-segregated with grain number/plot (Fig. 4b), fertile tillers/plot (Fig. 4c) and biomass (Fig. 4e) in three pairs of NIL (EK428_2, EK428_8 and EK664). NIL carrying the Excalibur allele showed 54.5% increase in grain yield, 43.5% in biomass and 32% in fertile tillers over the corresponding sibling line carrying the Kukri allele. NDVI significantly co-segregated with grain yield, grain number/plot, fertile tillers and biomass in EK428_2 and EK664 NIL. Kukri allele increased grain screenings in four NIL pairs (Fig. 4i). NIL with Kukri allele were characterized by many shrivelled grains as a result of severe drought and heat stress during grain filling stage.

By mapping the haplotypes of the five NIL against the DH genetic map and comparing their respective performance in semi-controlled field trial, we delimited *QYld.aww*-*1B.2* interval to 2.9 cM, flanked by the markers *BS00022342* and *ADW1218477* (Fig. 3c). This interval corresponds to 2.2 Mbp on chromosome 1B of the Chinese Spring reference sequence (Fig. 3c). Thirty-nine genes were predicted in the interval according to the IWGSC RefSeq v.1.0 annotation (Table 3).

**Table 3:** Description of the 39 predicted genes in the 2.2 Mbp interval between markers *adw1061145* and *adw572* containing *QYld.aww*-*1B.2*

IWGSC RefSeq v1.1 annotation	Description	Rice gene ID	*E*-value	Brachybodium gene ID	*E*-value
1 TraesCS1B02G437100	Calcium-transporting ATPase	LOC_Os05g49350.1	3.57E−39	Bradi2g45710.1	5.54E−29
2 TraesCS1B02G440100	Blue copper protein	LOC_Os05g49580.1	7.25E−62	Bradi2g15910.1	1.07E−69
3 TraesCS1B02G437600	Serine protease	LOC_Os05g49380.2	0	Bradi2g15980.1	0
4 TraesCS1B02G437200	F-box protein	LOC_Os07g35050.1	9.26E−57	Bradi2g37210.2	7.37E−105
5 TraesCS1B02G437500	Short-chain dehydrogenase/reductase	LOC_Os02g42810.1	5.51E−105	Bradi5g16660.1	7.65E−113
6 TraesCS1B02G440000	Blue copper protein	LOC_Os05g49580.1	3.06E−62	Bradi2g15920.1	3.33E−61
7 TraesCS1B02G440900	Mitochondrial transcription termination factor-like	LOC_Os06g12100.1	2.59E−52	Bradi3g00840.1	3.11E−66
8 TraesCS1B02G440500	WRKY transcription factor	LOC_Os05g49620.1	4.63E−59	Bradi2g15877.1	1.74E−55
9 TraesCS1B02G439600	Topoisomerase 1-associated factor 1	LOC_Os05g11980.1	0	Bradi2g15960.1	0
10 TraesCS1B02G437400	F-box protein	LOC_Os07g35060.1	1.99E−54	Bradi2g37210.2	1.42E−70
11 TraesCS1B02G439300	Disease resistance protein (TIR-NBS-LRR class) family	LOC_Os11g16470.1	5.65E−133	Bradi4g20527.5	1.18E−138
12 TraesCS1B02G438300	Disease resistance protein (NBS-LRR class) family	LOC_Os02g17304.1	4.72E−76	Bradi3g03882.1	8.52E−65
13 TraesCS1B02G438100	Disease resistance protein (NBS-LRR class) family	LOC_Os12g17420.1	5.13E−60	Bradi4g20527.5	1.41E−54
14 TraesCS1B02G440800	1-acyl-sn-glycerol-3-phosphate acyltransferase	LOC_Os05g49650.2	2.59E−69	Bradi2g15870.1	2.23E−66
15 TraesCS1B02G440200	Protein ROOT PRIMORDIUM DEFECTIVE 1	LOC_Os05g49610.1	0	Bradi2g15890.1	0
16 TraesCS1B02G438600	Disease resistance protein (TIR-NBS-LRR class) family	LOC_Os02g17304.1	0	Bradi4g04662.3	0
17 TraesCS1B02G437800	F-box family protein	LOC_Os07g18560.1	6.48E−87	Bradi2g16190.2	1.52E−82
18 TraesCS1B02G440400	Cation/H(+) antiporter	LOC_Os05g31730.1	2.88E−175	Bradi2g26740.1	0
19 TraesCS1B02G439500	F-box domain containing protein	LOC_Os03g24200.1	2.33E−87	Bradi4g42545.1	2.28E−144
20 TraesCS1B02G437300	F-box protein	LOC_Os05g08460.1	2.61E−29	Bradi2g37210.2	1.96E−51
21 TraesCS1B02G439900	Aldose 1-epimerase-like	LOC_Os05g49430.1	0	Bradi2g15930.1	0
22 TraesCS1B02G440300	WRKY transcription factor	LOC_Os05g49620.1	2.70E−57	Bradi2g15877.1	3.09E−61
23 TraesCS1B02G439800	G-box binding factor	LOC_Os05g49420.1	9.36E−92	Bradi2g15940.1	5.48E−98
24 TraesCS1B02G440700	WRKY transcription factor	LOC_Os05g49620.1	3.20E−54	Bradi2g15877.1	1.17E−54
25 TraesCS1B02G439700	tRNA dimethylallyltransferase	LOC_Os05g49410.1	6.78E−59	Bradi2g15950.1	8.42E−60
26 TraesCS1B02G441100	Mitochondrial transcription termination factor-like	LOC_Os06g12100.1	1.19E−54	Bradi1g58197.3	7.44E−68
27 TraesCS1B02G439400	Disease resistance protein (TIR-NBS-LRR class) family	LOC_Os02g17304.1	0	Bradi4g04662.3	0
28 TraesCS1B02G439200	Disease resistance protein RPP8	LOC_Os12g17480.1	0.00E+00	Bradi4g20527.4	0
29 TraesCS1B02G440600	WRKY transcription factor	LOC_Os05g49620.1	2.94E−44	Bradi2g15877.1	1.35E−47
30 TraesCS1B02G439000	Leucine-rich repeat receptor-like protein kinase family protein	LOC_Os02g06600.1	0.00E+00	Bradi3g04681.2	0
31 TraesCS1B02G438400	Disease resistance protein RPM1	LOC_Os11g16530.2	5.33E−17	Bradi4g04655.5	5.8E−11
32 TraesCS1B02G438800	Disease resistance protein (TIR-NBS-LRR class) family	LOC_Os02g17304.1	0.00E+00	Bradi4g20527.5	0
33 TraesCS1B02G438000	Leucine-rich repeat receptor-like protein kinase family protein	LOC_Os02g06600.1	2.03E−69	Bradi3g04710.1	4.9E−84
34 TraesCS1B02G438200	Disease resistance protein (TIR-NBS-LRR class)	LOC_Os08g29809.1	1.57E−61	Bradi4g20527.5	9.9E−61
35 TraesCS1B02G439100	Disease resistance protein (TIR-NBS-LRR class) family	LOC_Os02g17304.1	4.64E−129	Bradi4g04662.2	2.82E−122
36 TraesCS1B02G437900	Leucine-rich repeat receptor-like protein kinase family protein, putative	LOC_Os02g06600.1	2.25E−32	Bradi3g04681.1	2.08E−29
37 TraesCS1B02G437700	F-box family protein	LOC_Os07g18560.1	1.24E−130	Bradi2g16200.1	7.92E−121
38 TraesCS1B02G438500	Disease resistance protein (NBS-LRR class) family	LOC_Os12g17480.1	4.68E−180	Bradi4g20527.5	1.1E−180
39 TraesCS1B02G438900	F-box family protein	LOC_Os07g18560.1	3.10E−130	Bradi2g16200.1	2.17E−125

## Discussion

Grain yield in wheat has three major components: number of fertile spikes per area, number of grains per spike and TGW. Each component is controlled by multiple loci, which are often involved in complex interactions with each other and with the environment. In the present study, a total of 128 QTL were identified in Excalibur/Kukri DH population for four traits analysed across 32 experiments in three continents (Tables 1 and 2, Table S3). Among these QTL, 24 QTL for yield exhibited significant *Q* * *E* interactions; these QTL have the strongest allelic effect but are ‘unstable’ across environments. We also found 14 yield QTL with a significant but small, main effect across environments (Table 2).

As in the present study, several earlier studies identified grain yield QTL on the long arm of chromosome 1B (Bennett et al. 2012a, b; Graziani et al. 2014; Maccaferri et al. 2010; McIntyre et al. 2010; Shukla et al. 2015). *QYld.aww*-*1B.2* in Excalibur/Kukri DH population also coincided with a yield QTL in the RAC875/Kukri DH population found in multiple field trials from South Australia (Bennett et al. 2012b). Quarrie et al. (2005) found that the number of heads per plant, the number of grains per head and TGW were significantly associated with a grain yield QTL on chromosome 1B. Griffiths et al. (2015) also reported that the number of grains m^−2^ was significantly associated with the grain yield locus on chromosome 1B. Campbell et al. (1999) and Wu et al. (2015) also identified a TGW QTL in this region.

Surprisingly, we found large differences between alleles in four NIL pairs with the Excalibur allele increasing grain yield by 54.5%, biomass by 43.5% and fertile tillers by 32% over the Kukri allele. These are very large effects that are unlikely to be realized under farming conditions. Possible explanations for these large effects are: (1) we planted the NIL in August instead of the usual time of planting (May) which consequently exposed plants to extreme stresses; (2) plants had unlimited resources until stress which was imposed at heading. In a farming scenario, the plants are not equally spaced and are sown at approximately 200 seeds per m^2^. The plants were planted 10 cm apart in the polytunnel which favoured the expression of tiller number. These two conditions might have artificially exacerbated the QTL effects, with the serendipitous consequence of facilitating the QTL detection and fine mapping.

The co-segregation of grain yield with higher grain number/plot, fertile tillers, NDVI and biomass in four of the five NIL pairs (EK570, EK428_2, EK428_8 and EK664) (Fig. 3a–f) indicated that the differences in NDVI and fertile tillers might underlie the 1B yield QTL in Excalibur/Kukri. NDVI is a strong predictor of grain yield and is highly correlated with canopy biomass and early vigour (Gutiérrez-Rodríguez et al. 2004; Lukina et al. 1999; Raun et al. 2001; Tucker 1979). High early vigour or high NDVI contributes to high biomass accumulation and positively affects grain number m^−2^ and grain yield in wheat under conditions of terminal drought and heat (Foulkes et al. 2002). This is an important trait in the southern Australian environment where sufficient rainfall during the cool winter season is favourable for fast early biomass accumulation that could support carbohydrate supply to sink to maintain grain yield during terminal severe drought and high temperatures. Pre-anthesis assimilates not only contribute to grain/m^2^ but also to grain weight under terminal drought and heat (Rebetzke et al. 2008; Richards 1996; Yang et al. 2001).

A co-localization of QTL for NDVI, grain yield, grains/m^2^ and TGW on chromosome 1B under drought and heat stress was also reported earlier in the Seri/Babax population (Pinto et al. 2010). Similarly, a coincidence of QTL for NDVI with that for grain yield was also reported in RAC875/Kukri (Bennett et al. 2012a, b). It was suggested that QTL for NDVI are more closely associated with biomass production per se and greatly contribute to stem water soluble carbohydrates (WSC) that could be remobilized to developing grains under terminal drought stress (Ehdaie et al. 2008). WSC contribute up to 37–65% of grain yield under severe drought (Ehdaie et al. 2008). QTL for metabolite traits such as maltose and fructose were also reported on chromosome 1B in Excalibur/Kukri DH population under terminal drought and heat stress (Hill et al. 2013, 2015). These metabolites QTL had high expression in leaf under drought and heat stress and are the main components of WSC. Excalibur provided the positive allele for both traits. Interestingly, the maltose QTL (*QMal*-*1B* with flanking markers wPt0705 and wPt2526) was co-located with *QYld.aww*-*1B.2* in the Excalibur/Kukri DH population. This indicates that the *QYld.aww*-*1B.2* effects on yield might be due to the accumulation of maltose and fructose in leaves that could be translocated to grain during grain filling.

An increase in the number of grains per unit of land area is known to be partially offset by a reduction in grain weight (Slafer and Andrade 1993). This negative relationship between grain number and grain weight increases the proportion of small grains at particular positions of the spikelet and/or spike (Acreche and Slafer 2006; Slafer et al. 1999). TGW was not significantly different between NIL pairs in our study (Fig. 3j). This indicates that there was no significant compensation between TGW and number of grains in the environmental conditions we tested. Here, it is the number of fertile tillers that increased yield.

The higher number of tillers in the NIL carrying the Excalibur allele contributed to the higher number of grain number/plot by increasing fertile spikes/plot, thus leading to higher grain yield. de Oliveira et al. (2013) also reported an increase in grain yield under terminal drought and high temperatures with increased number of fertile tillers and grain number per unit area. The results of the present study and other studies elsewhere (e.g. Naruoka et al. 2011) indicate that plasticity in terms of fertile tillers per unit area is an important attribute for yield under drought and heat. Rapid ground cover with high tillering capacity enables cultivars to reduce soil water evaporation and increase light interception and assimilation capacity at pre-anthesis stage (Asseng and Van Herwaarden 2003; Blum 1997; Richards et al. 2002). Thus, increasing the number of fertile tillers per unit area would not necessarily reduce grain number and TGW, since the extra tillers would also increase stem carbohydrates and provide a source of assimilates for fertile spikes and grain weight during grain filling (Slafer et al. 1999). This might explain why there was no significant reduction in TGW associated with the increased number of fertile spikes and grain number/plot in the NIL (Fig. 3j). Further experiments will be required on source–sink relations and WSC status of the NIL to validate this hypothesis.

Grain yield between NIL for EK405 did not differ significantly, although they differ for the large QTL interval between 160.4 and 180.9 cM. The Excalibur allele of the QTL was associated with increased grain number per plot which could be explained, not by differences in tillering (there was no significant differences for number of fertile tillers per plant), but by a strong decrease in screenings. A possible explanation for the differences with the other NIL might be the differences in genetic background (Table S1). Even though the results of the genotyping of the NIL pairs using 22 genome-wide markers showed that they were mostly homogeneous, there might be other regions of the genome where they were segregating, and a more comprehensive marker genome coverage is needed to further assess this.

Although there are several examples of map-based cloning in wheat, no examples of cloning of QTL controlling grain yield under drought or heat stress are available. In the present study, we narrowed down *QYld.aww*-*1B.2* interval to a genomic region of 2.2 Mbp containing 39 predicted genes (Table 3). These predicted genes will need to be further studied to identify sequence variants that could explain the QTL in Excalibur/Kukri population. Sequence variations in intergenic regions should also be examined, since a previous study of the locus *teosinte branched1* (*tb1*) in maize demonstrated that the causal polymorphism might be in transposon element much upstream of the gene itself (Studer et al. 2011).

## Electronic supplementary material

Below is the link to the electronic supplementary material.

Supplementary material 1 (DOCX 65 kb)

Supplementary material 2 (PPTX 1383 kb)
