# Correction to: QTL analysis and fine mapping of a QTL for yield‑related traits in wheat grown in dry and hot environments

**DOI:** 10.1007/s00122-021-03792-4

**Published:** 2021-03-24

**Authors:** Habtamu Tura, James Edwards, Vijay Gahlaut, Melissa Garcia, Beata Sznajder, Ute Baumann, Fahimeh Shahinnia, Matthew Reynolds, Peter Langridge, Harindra Singh Balyan, Pushpendra K. Gupta, Thorsten Schnurbusch, Delphine Fleury

**Affiliations:** 1 School of Agriculture, Food and Wine, Waite Campus, University of Adelaide, PMB1, Glen Osmond, SA 5064 Australia; 2 Australian Grain Technologies, 20 Leitch Road, Roseworthy, SA Australia; 3 Department of Genetics and Plant Breeding, Ch. Charan Singh University, Meerut, India; 4 Present Address: Institute for Crop Science and Plant Breeding, Bavarian State Research Center for Agriculture, Am Gereuth 8, 85354 Freising, Germany; 5 International Maize and Wheat Improvement Center (CIMMYT), Int. AP 6-641, 06600 Mexico, D.F. Mexico; 6 Julius-Kühn-Institute, Königin-Louise-Str 19, 14195 Berlin, Germany; 7 Present Address: Leibniz-Institute of Plant Genetics and Crop Plant Research (IPK), Corrensstr. 3, 06466 Gatersleben, Germany

## Correction to: Theoretical and Applied Genetics (2020) 133:239–257 https://doi.org/10.1007/s00122-019-03454-6

The article “QTL analysis and fine mapping of a QTL for yield‑related traits in wheat grown in dry and hot environments”, written by Melissa Garcia, was originally published Online First without Open Access. After publication in volume 133, issue 1, page 239–257 the author decided to opt for Open Choice and to make the article an Open Access publication. Therefore, the copyright of the article has been changed to © The Author(s) 2020 and this article is licensed under a Creative Commons Attribution 4.0 International License, which permits use, sharing, adaptation, distribution and reproduction in any medium or format, as long as you give appropriate credit to the original author(s) and the source, provide a link to the Creative Commons licence, and indicate if changes were made. The images or other third party material in this article are included in the article's Creative Commons licence, unless indicated otherwise in a credit line to the material. If material is not included in the article's Creative Commons licence and your intended use is not permitted by statutory regulation or exceeds the permitted use, you will need to obtain permission directly from the copyright holder. To view a copy of this licence, visit http://creativecommons.org/licenses/by/4.0/.

The original article has been corrected.

**Open Access** This article is licensed under a Creative Commons Attribution 4.0 International License, which permits use, sharing, adaptation, distribution and reproduction in any medium or format, as long as you give appropriate credit to the original author(s) and the source, provide a link to the Creative Commons licence, and indicate if changes were made. The images or other third party material in this article are included in the article's Creative Commons licence, unless indicated otherwise in a credit line to the material. If material is not included in the article's Creative Commons licence and your intended use is not permitted by statutory regulation or exceeds the permitted use, you will need to obtain permission directly from the copyright holder. To view a copy of this licence, visit http://creativecommons.org/licenses/by/4.0/.

