# Supplementary material for: QTL analysis and fine mapping of a QTL for yield-related traits in wheat grown in dry and hot environments
Source: Theor Appl Genet. 2019 Oct 4;133(1):239–57. doi: 10.1007/s00122-019-03454-6 (PMC7990757; doi:10.1007/s00122-019-03454-6)
Supplement: Supplementary file 1 — Supplementary material 1 (DOCX 65 kb) [file 122_2019_3454_MOESM1_ESM.docx]

Supplementary Table S1 Genotype of the six NIL pairs for eight SNP KASP™ markers spanning the region of *QYld.aww-1B.2* and 22 markers distributed across 17 chromosomes. - means missing value. Het means heterozygous. Chr means Chromosome; NA means that the BLAST results were not conclusive.

| Markers/lines | Chr | Position in the physical map* | Excalibur | Kukri | EK405_AA | EK405_BB | EK428_2AA | EK428_8AA | EK428_BB | EK570_AA | EK570_BB | EK664_AA | EK664_BB |
| --- | --- | --- | --- | --- | --- | --- | --- | --- | --- | --- | --- | --- | --- |
| BS00005272 | 1A | 1159630 - 1159749 | AA | BB | AA | AA | AA | AA | AA | BB | BB | BB | BB |
| BS00012226 | 1D | 10660412 - 10660932 | AA | BB | AA | AA | AA | AA | AA | BB | BB | BB | BB |
| BS00022707 | 2A | 650629871 - 650629925 | AA | BB | AA | AA | BB | BB | BB | AA | AA | AA | AA |
| BS00072058 | 2B | 12877086 - 12877186 | AA | BB | BB | AA | AA | AA | AA | BB | BB | BB | BB |
| BS00023317 | 2D | 73312478 - 73312754 | AA | BB | AA | AA | BB | BB | BB | BB | BB | BB | BB |
| BS00025739 | 3A | 25939213 - 25939313 | AA | BB | AA | AA | AA | AA | AA | BB | BB | BB | BB |
| BS00022401 | 3A | 743757888 - 743757949 | AA | BB | AA | AA | BB | BB | BB | AA | AA | AA | AA |
| BS00089651 | 3B | 23600367 - 23600467 | AA | BB | AA | AA | AA | AA | AA | BB | BB | BB | BB |
| BS00076816 | 3B | 56340242 - 56340342 | AA | BB | AA | AA | AA | AA | AA | AA | AA | BB | BB |
| BS00067015 | 3D | 558566167 - 558566219 | AA | BB | BB | BB | BB | BB | BB | BB | BB | BB | BB |
| BS00022125 | 4A | 43698633 - 43699147 | AA | BB | AA | AA | AA | AA | AA | AA | AA | BB | BB |
| BS00021738 | 4A | 596304943 - 596305356 | AA | BB | BB | BB | AA | AA | AA | AA | AA | BB | BB |
| BS00021727 | 4A | 606732994 - 606733488 | AA | BB | AA | AA | AA | AA | AA | AA | AA | BB | BB |
| BS00011510 | 4B | 16056588 - 16056759 | AA | BB | BB | BB | BB | - | BB | AA | AA | BB | BB |
| BS00021955 | 5A | 559516762 - 559517121 | AA | BB | BB | BB | AA | AA | AA | AA | AA | AA | AA |
| BS00016003 | 5B | 53024026 - 53024079 | AA | BB | AA | AA | AA | AA | AA | BB | BB | AA | AA |
| BS00009821 | 5D | 550509799 - 550509842 | AA | BB | AA | AA | AA | AA | AA | AA | AA | AA | AA |
| BS00021982 | 6A | 5604230 - 5604466 | AA | BB | - | - | AA | AA | AA | BB | BB | BB | BB |
| BS00181492 | 6B | 4876497 - 4876552 | AA | BB | AA | AA | AA | AA | AA | BB | BB | BB | BB |
| BS00022204 | 6D | 465956898 - 465957989 | AA | BB | AA | AA | BB | BB | BB | AA | AA | Het | AA |
| BS00089134 | 7A | 125260206 - 125260306 | AA | BB | AA | AA | AA | AA | AA | AA | AA | BB | BB |
| BS00009290 | 7B | 640717215 - 640717265 | AA | BB | AA | AA | BB | BB | BB | BB | BB | BB | BB |
| ADW1061145 | 1B | 644598782 - 644598850 | AA | BB | AA | AA | AA | AA | AA | BB | BB | AA | AA |
| BS00066864 | 1B | 652575151 - 652575212 | AA | BB | AA | BB | AA | AA | AA | BB | BB | AA | BB |
| ADW1218477 | 1B | 659985890 - 659985773 | AA | BB | AA | BB | AA | AA | BB | AA | BB | AA | BB |
| BS00022342 | 1B | 662154382 - 662154451 | AA | BB | AA | BB | AA | AA | BB | AA | BB | AA | BB |
| adw525 | 1B | 667966017 - 667966217 | AA | BB | AA | BB | AA | BB | BB | AA | BB | AA | BB |
| adw535 | 1B | 670173154 - 670173354 | AA | BB | AA | BB | AA | BB | BB | AA | BB | AA | BB |
| BS00021877 | 1B | 673742912 - 673742972 | AA | BB | AA | BB | BB | BB | BB | AA | BB | AA | BB |
| adw572 | 1B | NA | AA | BB | BB | BB | BB | BB | BB | AA | BB | BB | BB |
| *Position of SNP probe in the CS IWGSC RefSeq v.1 obtained through URGI or BLASTN. | | | | | | | | |  |  |  |  |  |

**Supplementary Table S2**. Field experiments, location and abbreviation details.

| **Trial** | **Address** | **Latitude** | **Longitude** | **Altitude (m)** |
| --- | --- | --- | --- | --- |
| Boo | Booleroo Centre, South Australia, Australia | 32.88^o^S | 138.35^o^E | 390 |
| Min | Minnipa Agricultural Research Centre, Minnipa, South Australia, Australia | 32.86^o^S | 135.14^o^E | 139 |
| Pie | Piednippie, South Australia, Australia | 32.44^o^S | 134.28^o^E | 85 |
| Ros | University of Adelaide, Roseworthy Campus, Roseworthy, South Australia, Australia | 34.57^o^S | 138.74^o^E | 87 |
| Rob | Robinvale, Victoria, Australia | 34.59^o^S | 142.78^o^E | 58 |
| Obr | International Maze and Wheat Improvement Centre, CIANO, Obregon, Mexico | 27.20^o^N | 109.56^o^W | 38 |
| His | Chaudhary Charan Singh Haryana Agricultural University, Hisar, Haryana, India | 29.15^o^N | 75.70^o^E | 215 |
| Kan | Chandra Shekhar Azad University of Agriculture and Technology, Kanpur, Uttar Pradesh, India | 26.27^o^N | 80.14^o^E | 126 |
| Kar | Indian Institute of Wheat and Barley Research, Karnal, Haryana, India | 29.68^o^N | 76.98^o^E | 227 |
| Pun | Agharkar Research Institute, Pune, Maharashtra,  India | 18.31^o^N | 73.52^o^E | 560 |

**Supplementary Table S3** Allelic QTL effects for days to heading and grain filling duration. P value is derived from the Wald test for the effect indicated by Term (QTL main effect or QTL by environment interaction Q * E). QTL effect (in days) provides the estimate of the effect indicated by Term, with a range of the estimates per environment that were significantly different from zero shown for Q * E effect (confidence interval of the estimate calculated as CI = estimate +/- 1.96*SE). Positive number shows allelic effect from Excalibur, negative number from Kukri.

| **Chr** | **QTL** | **Flanking markers** | **QTL peak**  **(cM)** | **Term** | **P value** | **QTL effect** |
| --- | --- | --- | --- | --- | --- | --- |
| **Days to heading** | |  |  |  |  |  |
| 1D | *QDth.aww-1D* | X2243386-1126683 | 70.3-84.1 | Main effect | <0.001 | -0.8 |
| 2A | *QDth.aww-2A* | X1008748-X100003139 | 33-39.3 | Main effect | <0.01 | 0.6 |
| 2B | *QDth.aww-2B* | X3064555-X981297 | 53.5-54.2 | Q * E | <0.01 | -4 to -2 |
| 2D | *QDth.aww-2D.1* | wPt-3728-wPt-0638 | 38.9-40 | Q * E | <0.01 | -1 to 2 |
| 2D | *QDth.aww-2D.2* | X1046316-wPt-1991 | 70.5-74.4 | Main effect | <0.01 | 0.8 |
| 3A | *QDth.aww-3A.1* | wPt-4407-X2266014 | 39-45.6 | Main effect | <0.001 | 0.8 |
| 3A | *QDth.aww-3A.2* | X1132581-X1014677 | 85.8-86.5 | Q * E | <0.01 | -2 to 2 |
| 3A | *QDth.aww-3A.3* | X1121175-cfa2170 | 114.2-123.2 | Main effect | <0.0001 | 1 |
| 3DSL | *QDth.aww-3DSL.1* | wPt-2313-2245896 | 7.4-22 | Q * E | <0.001 | 3 to 3 |
| 3DSL | *QDth.aww-3DSL.2* | wPt-6262-X1125700 | 59-60.4 | Main effect | <0.01 | -0.9 |
| 4A | *QDth.aww-4A.1* | X1130647-X1242399 | 124-124.4 | Main effect | <0.001 | 0.7 |
| 4A | *QDth.aww-4A.2* | X3064552-X1125529 | 130-142.7 | Q * E | <0.01 | 2 to 5 |
| 4B | *QDth.aww-4B* | wPt-1272-wPt-0246 | 76.7-77.3 | Main effect | <0.001 | 0.7 |
| 4D | *QDth.aww-4D* | X1004846-X1161775 | 35.2-37.8 | Main effect | <0.01 | 0.9 |
| 5A | *QDth.aww-5A* | X1135154-Vrn1A | 123.6-125.5 | Q * E | <0.0001 | 2 to 7 |
| 5B | *QDth.aww-5B.1* | wPt-1733-1055936 | 84.8-86.1 | Main effect | <0.001 | 0.8 |
| 5B | *QDth.aww-5B.2* | X1104787-X1030395 | 109.5-109.6 | Q * E | <0.01 | -3 to 1 |
| 5B | *QDth.aww-5B.3* | X1240475-X3029964 | 161.5-172.7 | Q * E | <0.01 | -2 to 3 |
| 6A | *QDth.aww-6A.1* | X1128290-X1247837 | 12.3-13.6 | Main effect | <0.001 | -1 |
| 6A | *QDth.aww-6A.2* | wPt-9474-wPt-9976 | 99.6-100.3 | Main effect | <0.01 | 1 |
| 6B | *QDth.aww-6B* | X1098568-WPT-1048 | 120.4-123.1 | Main effect | <0.01 | 0.7 |
| 7A | *QDth.aww-7A.1* | 1246868-X1228158 | 56.3-56.9 | Q * E | <0.0001 | -13 to -2 |
| 7A | *QDth.aww-7A.2* | barc0195-X1127751.6TC | 95.5-97.4 | Main effect | <0.001 | -1 |
| 7B | *QDth.aww-7B.1* | X1008572-X1712267 | 57.6-59 | Main effect | <0.001 | 0.7 |
| 7B | *QDth.aww-7B.1* | X2281290-X1049564 | 85.2-85.8 | Main effect | <0.01 | 0.7 |
| 7B | *QDth.aww-7B.2* | BSm3603_7B-wPt-6156 | 109.9-110.6 | Main effect | <0.01 | 0.7 |
| 7DS | *QDth.aww-7DS* | WPT-0366-WPT-5049 | 1.1-1.8 | Q * E | <0.05 | -2 to 1 |
| **Grain filling duration** | |  |  |  |  |  |
| 1A | *QGfd.aww-1A* | X100000274-X1023638 | 0-0.6 | Main effect | <0.01 | 0.1 |
| 1B | *QGfd.aww-1B* | wmc0830-psp3100 | 105.3-110.5 | Main effect | <0.01 | -0.2 |
| 2B | *QGfd.aww-2B.1* | X3021359-X3064555 | 52.9-53.5 | Main effect | <0.001 | -0.1 |
| 2B | *QGfd.aww-2B.2* | X1025310-X1017398 | 65.3-65.9 | Q * E | <0.01 | -2 to 1 |
| 2B | *QGfd.aww-2B.3* | X1143915-X1133994 | 85.5-87.4 | Main effect | <0.001 | -0.2 |
| 2B | *QGfd.aww-2B.4* | X1138420-X1160435 | 106.6-109.8 | Q * E | <0.01 | -2 to 1 |
| 2D | *QGfd.aww-2D* | X2242328-X991014 | 19.4-28.2 | Q * E | <0.01 | -2 to 2 |
| 3A | *QGfd.aww-3A.1* | wPt-9369-X1259016 | 11.2-14.2 | Main effect | <0.01 | -0.2 |
| 3A | *QGfd.aww-3A.2* | X3022477-X2294497 | 48-52.6 | Main effect | <0.01 | -0.2 |
| 3A | *QGfd.aww-3A.3* | X2254081-X1086149 | 63.3-64.3 | Q * E | <0.01 | -2 to -1 |
| 3A | *QGfd.aww-3A.4* | X1132581-X1014677 | 85.8-86.5 | Main effect | <0.001 | -0.2 |
| 3B | *QGfd.aww-3B.1* | X1032586-wPt-8206 | 191.7-194.6 | Main effect | <0.001 | 0.3 |
| 3B | *QGfd.aww-3B.2* | cfb577-X984052 | 205.9-206.4 | Q * E | <0.001 | -2 to 2 |
| 4A | *QGfd.aww-4A* | X3029395-X1064602 | 9.8-25.5 | Q * E | <0.01 | -1 to 2 |
| 4B | *QGfd.aww-4B.1* | wPt-3608-wmc0047 | 12.7-22.1 | Main effect | <0.0001 | 0.1 |
| 4B | *QGfd.aww-4B.2* | gwm0495-ksm0154 | 43.4-45.5 | Main effect | <0.0001 | 0.1 |
| 4B | *QGfd.aww-4B.3* | wPt-0246-wPt-8650 | 77.3-81.7 | Main effect | <0.01 | 0.2 |
| 4D | *QGfd.aww-4D* | X1065922-X2256312 | 0-8 | Main effect | <0.01 | 0 |
| 5A | *QGfd.aww-5A.1* | wPt-6048-X1215677 | 0-0.6 | Q * E | <0.01 | -1 to 2 |
| 5A | *QGfd.aww-5A.2* | X1135154-Vrn1A | 123.6-125.5 | Q * E | <0.0001 | -3 to -2 |
| 6A | *QGfd.aww-6A* | X1397091-wPt-8006 | 0-6.5 | Main effect | <0.01 | 0.3 |
| 6B | *QGfd.aww-6B* | wPt-5408-X3020939 | 39.9-40 | Q * E | <0.001 | -1 to 1 |
| 7A | *QGfd.aww-7A* | 1246868-X1228158.44AG | 56.3-56.9 | Q * E | <0.0001 | -1 to 4 |
| 7B | *QGfd.aww-7B* | X987292-X1083752 | 6.8-29.2 | Q * E | <0.01 | -1 to 2 |
| 7DSL | *QGfd.aww-7DSL* | stm0789tcacD-WPT-0789 | 21.1-29.1 | Main effect | <0.01 | 0 |

**Supplementary Table S4** Correlations among yield and yield components in Excalibur/Kukri NIL under severe drought and heat condition (Urrbrae, South Australia, 2016).

BM = biomass

TGN = total grain number per plot

GY = grain yield

FT = fertile tillers

GS = grain/spike

GSp = grain/spikelet

TGW = thousand grain weight

SpS = Spikelet/Spike

NDVI = Normalize difference vegetative index

PH = plant height

SL = spike length

Scr = Screening (%)

Values are Pearson correlation coefficients, with significance levels indicated by:

p <0.01

p < 0.001
