# Supplementary material for: QTL analysis and fine mapping of a QTL for yield-related traits in wheat grown in dry and hot environments
Source: Theor Appl Genet. 2019 Oct 4;133(1):239–57. doi: 10.1007/s00122-019-03454-6 (PMC7990757; doi:10.1007/s00122-019-03454-6)
Supplement: Supplementary file 2 — Supplementary material 2 (PPTX 1383 kb) [file 122_2019_3454_MOESM2_ESM.pptx]

## Slide 1
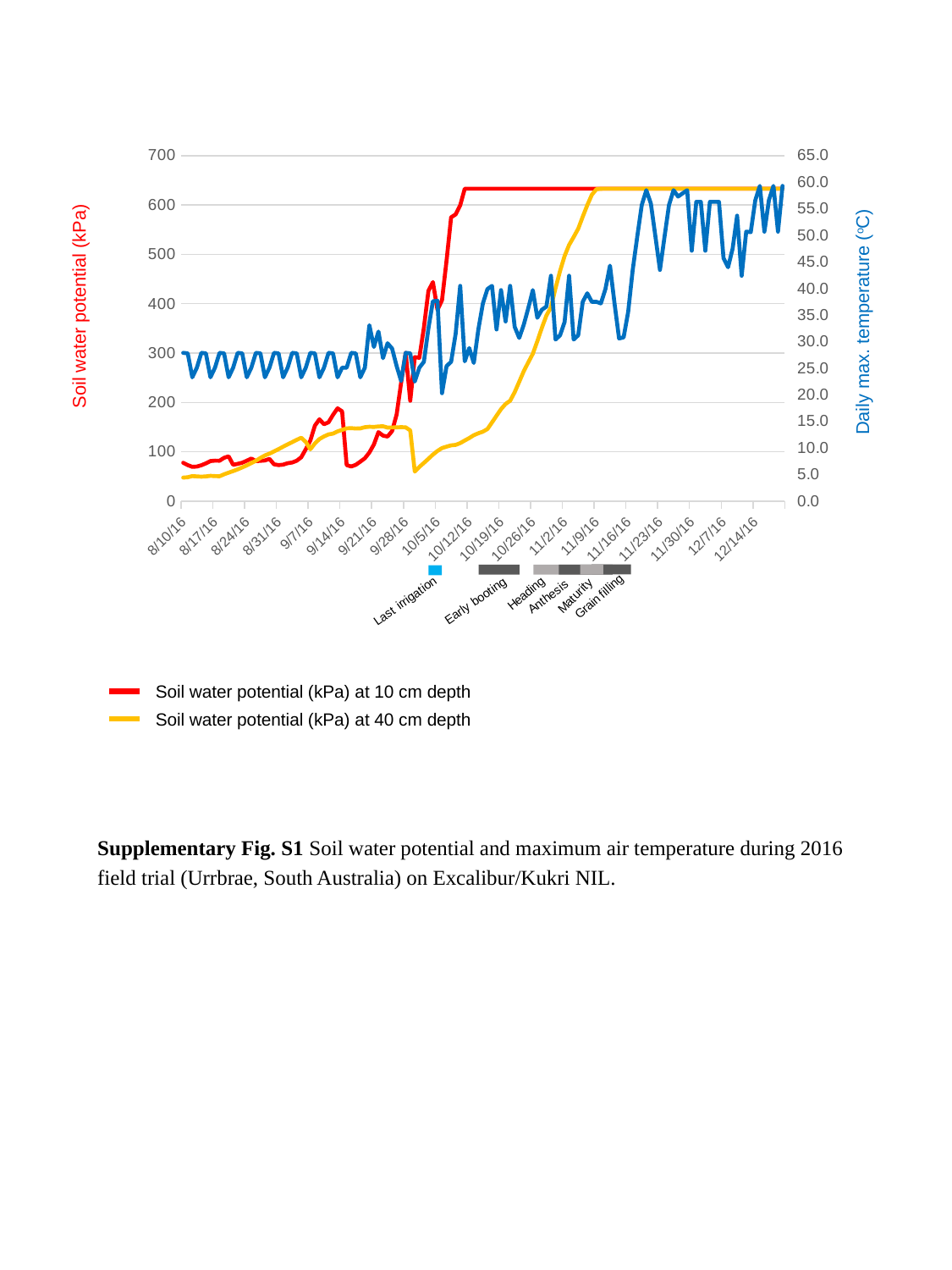

### Chart
| Category | Soil-water-tension(Kpa)_10 cM | Soil-water-tension(Kpa)_40 cM | Max. temp (0C) |
|---|---|---|---|
| 42592 | 77.59722222222223 | 47.541666666666664 | 27.9 |
| 42593 | 72.9375 | 48.30555555555556 | 27.8 |
| 42594 | 69.30555555555556 | 50.55555555555556 | 23.3 |
| 42595 | 69.95833333333333 | 50.0 | 25.1 |
| 42596 | 72.60416666666667 | 49.520833333333336 | 27.9 |
| 42597 | 76.43055555555556 | 49.979166666666664 | 27.8 |
| 42598 | 81.09722222222223 | 51.083333333333336 | 23.3 |
| 42599 | 81.95138888888889 | 50.63194444444444 | 25.1 |
| 42600 | 81.67361111111111 | 50.31944444444444 | 27.9 |
| 42601 | 87.71527777777777 | 54.25694444444444 | 27.8 |
| 42602 | 90.625 | 57.56944444444444 | 23.3 |
| 42603 | 73.5 | 60.77777777777778 | 25.1 |
| 42604 | 75.41666666666667 | 64.24305555555556 | 27.9 |
| 42605 | 77.75 | 68.25 | 27.8 |
| 42606 | 81.77083333333333 | 72.23611111111111 | 23.3 |
| 42607 | 86.1875 | 76.94444444444444 | 25.1 |
| 42608 | 80.97222222222223 | 81.75 | 27.9 |
| 42609 | 81.48611111111111 | 87.42361111111111 | 27.8 |
| 42610 | 82.26388888888889 | 92.43055555555556 | 23.3 |
| 42611 | 85.47222222222223 | 96.22222222222223 | 25.1 |
| 42612 | 74.49305555555556 | 100.49305555555556 | 27.9 |
| 42613 | 72.84722222222223 | 105.20138888888889 | 27.8 |
| 42614 | 73.78472222222223 | 110.11805555555556 | 23.3 |
| 42615 | 76.76388888888889 | 114.90277777777777 | 25.1 |
| 42616 | 78.125 | 119.34027777777777 | 27.9 |
| 42617 | 81.79166666666667 | 124.13194444444444 | 27.8 |
| 42618 | 88.78472222222223 | 128.25694444444446 | 23.3 |
| 42619 | 105.07638888888889 | 119.74305555555556 | 25.1 |
| 42620 | 122.72222222222223 | 104.86805555555556 | 27.9 |
| 42621 | 152.85416666666666 | 116.85416666666667 | 27.8 |
| 42622 | 165.65277777777777 | 125.61805555555556 | 23.3 |
| 42623 | 155.65972222222223 | 130.9375 | 25.1 |
| 42624 | 159.63888888888889 | 135.125 | 27.9 |
| 42625 | 174.70138888888889 | 136.75 | 27.8 |
| 42626 | 188.11111111111111 | 141.21527777777777 | 23.3 |
| 42627 | 181.66666666666666 | 144.23611111111111 | 25.1 |
| 42628 | 72.88888888888889 | 147.3125 | 25.1 |
| 42629 | 70.04166666666667 | 147.79166666666666 | 27.9 |
| 42630 | 73.40972222222223 | 147.06944444444446 | 27.8 |
| 42631 | 79.82638888888889 | 147.22916666666666 | 23.3 |
| 42632 | 86.75 | 149.64583333333334 | 25.1 |
| 42633 | 98.27083333333333 | 150.61805555555554 | 33.1 |
| 42634 | 114.76388888888889 | 150.22222222222223 | 29.0 |
| 42635 | 139.64583333333334 | 151.14583333333334 | 31.9 |
| 42636 | 132.64583333333334 | 151.59722222222223 | 26.9 |
| 42637 | 130.74305555555554 | 148.89583333333334 | 29.7 |
| 42638 | 141.81944444444446 | 149.1875 | 28.7 |
| 42639 | 175.53472222222223 | 149.39583333333334 | 25.4 |
| 42640 | 239.98611111111111 | 149.74305555555554 | 22.6 |
| 42641 | 300.3333333333333 | 149.38888888888889 | 27.9 |
| 42642 | 203.35872758194188 | 143.23316713048857 | 27.8 |
| 42643 | 291.8125 | 59.68055555555556 | 22.5 |
| 42644 | 290.34027777777777 | 69.14583333333333 | 25.1 |
| 42645 | 351.2708333333333 | 77.35416666666667 | 26.2 |
| 42646 | 426.0208333333333 | 85.60416666666667 | 32.5 |
| 42647 | 443.7361111111111 | 94.11111111111111 | 37.6 |
| 42648 | 387.3888888888889 | 101.56944444444444 | 37.7 |
| 42649 | 407.03472222222223 | 107.44444444444444 | 20.3 |
| 42650 | 487.5 | 110.29166666666667 | 25.4 |
| 42651 | 575.0555555555555 | 112.88888888888889 | 26.2 |
| 42652 | 580.8888888888889 | 113.79166666666667 | 31.5 |
| 42653 | 599.3819444444445 | 117.44444444444444 | 40.5 |
| 42654 | 632.9444444444445 | 122.69444444444444 | 26.3 |
| 42655 | 633.0 | 127.71527777777777 | 28.8 |
| 42656 | 633.0 | 133.46527777777777 | 26.0 |
| 42657 | 633.0 | 137.38194444444446 | 32.3 |
| 42658 | 633.0 | 140.61805555555554 | 37.2 |
| 42659 | 633.0 | 145.93055555555554 | 39.9 |
| 42660 | 633.0 | 159.27083333333334 | 40.5 |
| 42661 | 633.0 | 172.93055555555554 | 32.3 |
| 42662 | 633.0 | 186.27777777777777 | 39.7 |
| 42663 | 633.0 | 196.86805555555554 | 33.8 |
| 42664 | 633.0 | 203.36805555555554 | 40.5 |
| 42665 | 633.0 | 220.64583333333334 | 32.8 |
| 42666 | 633.0 | 241.90972222222223 | 30.7 |
| 42667 | 633.0 | 263.2986111111111 | 33.2 |
| 42668 | 633.0 | 281.0208333333333 | 36.3 |
| 42669 | 633.0 | 298.5486111111111 | 39.7 |
| 42670 | 633.0 | 323.9513888888889 | 34.5 |
| 42671 | 633.0 | 351.125 | 36.0 |
| 42672 | 633.0 | 376.0208333333333 | 36.6 |
| 42673 | 633.0 | 392.7916666666667 | 42.4 |
| 42674 | 633.0 | 431.21527777777777 | 30.4 |
| 42675 | 633.0 | 466.2916666666667 | 31.2 |
| 42676 | 633.0 | 496.63194444444446 | 33.7 |
| 42677 | 633.0 | 519.0069444444445 | 42.4 |
| 42678 | 633.0 | 535.0486111111111 | 30.4 |
| 42679 | 633.0 | 551.8680555555555 | 31.2 |
| 42680 | 633.0 | 576.3055555555555 | 37.5 |
| 42681 | 633.0 | 599.6805555555555 | 39.1 |
| 42682 | 633.0 | 620.6180555555555 | 37.5 |
| 42683 | 633.0 | 631.8333333333334 | 37.5 |
| 42684 | 633.0 | 632.9236111111111 | 37.2 |
| 42685 | 633.0 | 633.0 | 39.9 |
| 42686 | 633.0 | 633.0 | 44.3 |
| 42687 | 633.0 | 633.0 | 37.2 |
| 42688 | 633.0 | 633.0 | 30.6 |
| 42689 | 633.0 | 633.0 | 30.8 |
| 42690 | 633.0 | 633.0 | 35.6 |
| 42691 | 633.0 | 633.0 | 43.5 |
| 42692 | 633.0 | 633.0 | 49.7 |
| 42693 | 633.0 | 633.0 | 55.7 |
| 42694 | 633.0 | 633.0 | 58.5 |
| 42695 | 633.0 | 633.0 | 56.0 |
| 42696 | 633.0 | 633.0 | 49.8 |
| 42697 | 633.0 | 633.0 | 43.5 |
| 42698 | 633.0 | 633.0 | 49.7 |
| 42699 | 633.0 | 633.0 | 55.7 |
| 42700 | 633.0 | 633.0 | 58.5 |
| 42701 | 633.0 | 633.0 | 57.3 |
| 42702 | 633.0 | 633.0 | 57.9 |
| 42703 | 633.0 | 633.0 | 58.5 |
| 42704 | 633.0 | 633.0 | 47.1 |
| 42705 | 633.0 | 633.0 | 56.3 |
| 42706 | 633.0 | 633.0 | 56.3 |
| 42707 | 633.0 | 633.0 | 47.1 |
| 42708 | 633.0 | 633.0 | 56.3 |
| 42709 | 633.0 | 633.0 | 56.3 |
| 42710 | 633.0 | 633.0 | 56.3 |
| 42711 | 633.0 | 633.0 | 45.7 |
| 42712 | 633.0 | 633.0 | 44.0 |
| 42713 | 633.0 | 633.0 | 47.5 |
| 42714 | 633.0 | 633.0 | 53.7 |
| 42715 | 633.0 | 633.0 | 42.4 |
| 42716 | 633.0 | 633.0 | 50.7 |
| 42717 | 633.0 | 633.0 | 50.6 |
| 42718 | 633.0 | 633.0 | 56.6 |
| 42719 | 633.0 | 633.0 | 59.3 |
| 42720 | 633.0 | 633.0 | 50.7 |
| 42721 | 633.0 | 633.0 | 56.6 |
| 42722 | 633.0 | 633.0 | 59.3 |
| 42723 | 633.0 | 633.0 | 50.7 |
| 42724 | 633.0 | 633.0 | 59.3 |Soil water potential (kPa)
Daily max. temperature (oC)
Soil water potential (kPa) at 10 cm depth
Soil water potential (kPa) at 40 cm depth
Supplementary Fig. S1 Soil water potential and maximum air temperature during 2016 field trial (Urrbrae, South Australia) on Excalibur/Kukri NIL.

## Slide 2
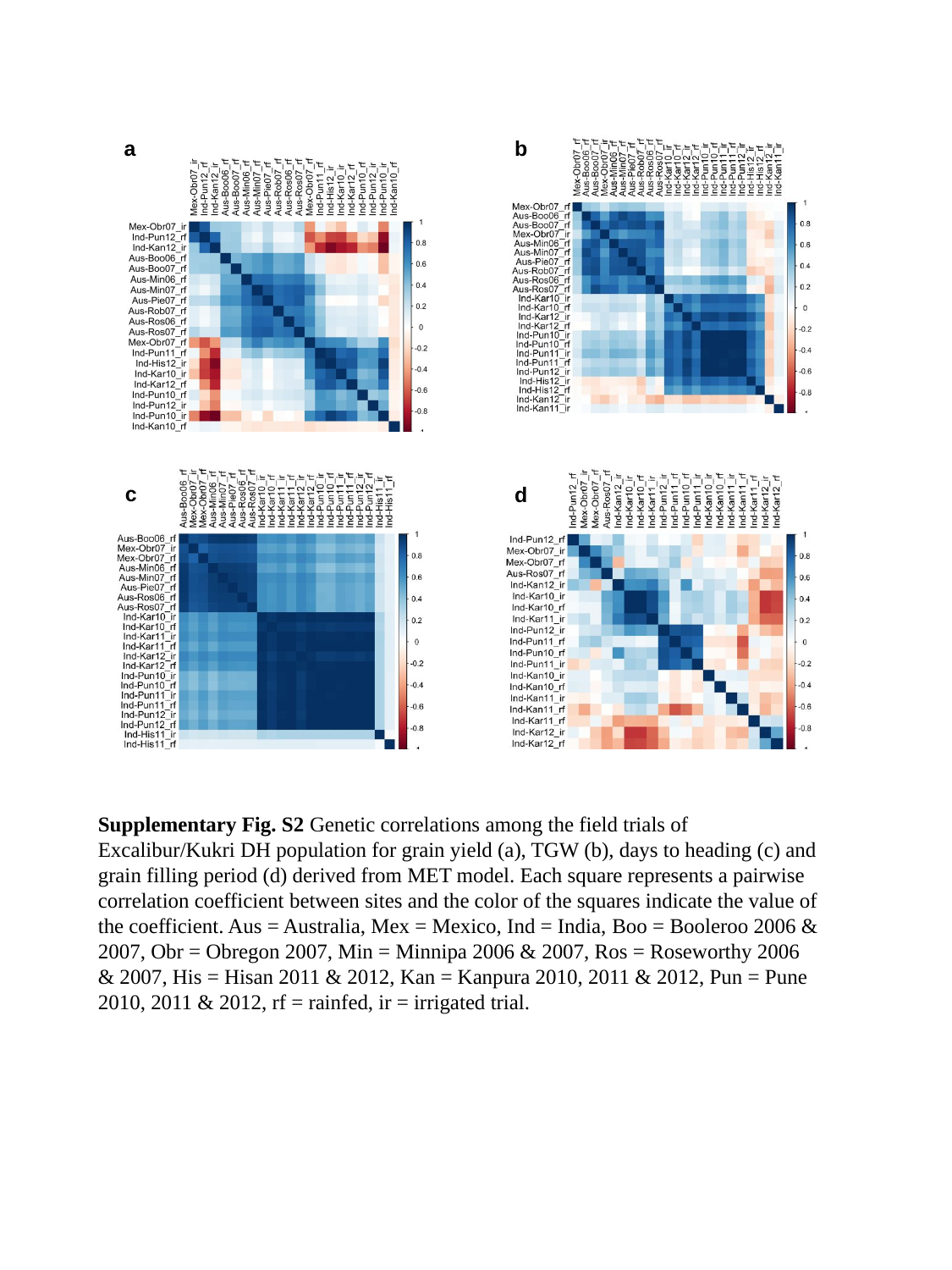

a
b
c
d
Supplementary Fig. S2 Genetic correlations among the field trials of Excalibur/Kukri DH population for grain yield (a), TGW (b), days to heading (c) and grain filling period (d) derived from MET model. Each square represents a pairwise correlation coefficient between sites and the color of the squares indicate the value of the coefficient. Aus = Australia, Mex = Mexico, Ind = India, Boo = Booleroo 2006 & 2007, Obr = Obregon 2007, Min = Minnipa 2006 & 2007, Ros = Roseworthy 2006 & 2007, His = Hisan 2011 & 2012, Kan = Kanpura 2010, 2011 & 2012, Pun = Pune 2010, 2011 & 2012, rf = rainfed, ir = irrigated trial.

## Slide 3
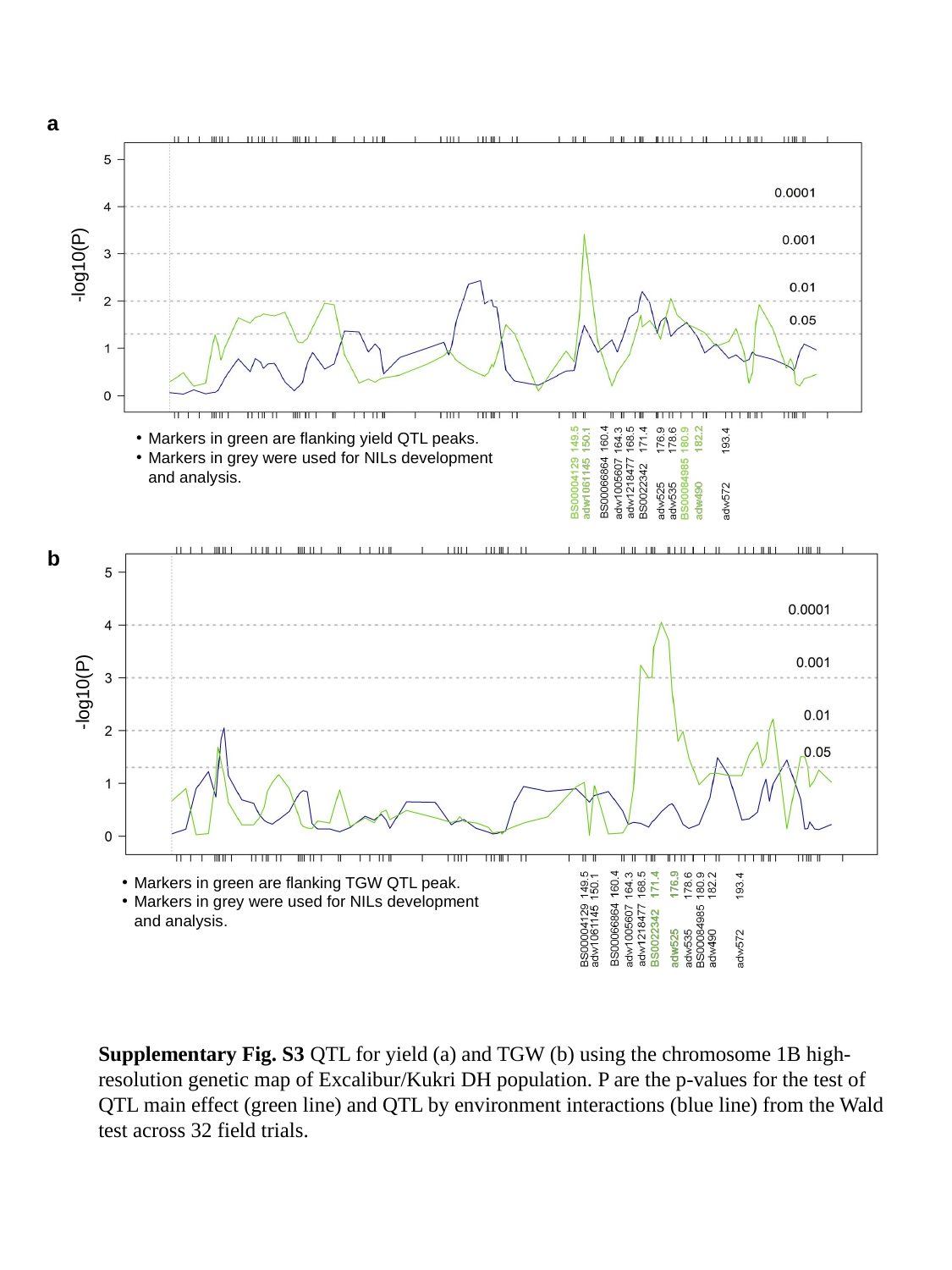

a
Markers in green are flanking yield QTL peaks.
Markers in grey were used for NILs development and analysis.
-log10(P)
b
Markers in green are flanking TGW QTL peak.
Markers in grey were used for NILs development and analysis.
-log10(P)
Supplementary Fig. S3 QTL for yield (a) and TGW (b) using the chromosome 1B high-resolution genetic map of Excalibur/Kukri DH population. P are the p-values for the test of QTL main effect (green line) and QTL by environment interactions (blue line) from the Wald test across 32 field trials.
